# The Evolving Landscape of Novel and Old Biomarkers in Localized High-Risk Prostate Cancer: State of the Art, Clinical Utility, and Limitations Toward Precision Oncology

**DOI:** 10.3390/jpm15080367

**Published:** 2025-08-11

**Authors:** Lilia Bardoscia, Angela Sardaro, Mariagrazia Quattrocchi, Paola Cocuzza, Elisa Ciurlia, Ilaria Furfaro, Maria Antonietta Gilio, Marcello Mignogna, Beatrice Detti, Gianluca Ingrosso

**Affiliations:** 1 Radiation Oncology Unit, Oncology Department, S. Luca Hospital, Azienda USL Toscana Nord Ovest, 55100 Lucca, Italy; 2 Radiation Oncology Unit, Vito Fazzi Hospital, 73100 Lecce, Italy; 3 Medical School, University of Bari “Aldo Moro”, 70124 Bari, Italy; 4 Medical Physics Department, Azienda USL Toscana Nord Ovest, 55100 Lucca, Italy; 5 Medical Oncology Unit, Oncology Department, S. Luca Hospital, Azienda USL Toscana Nord Ovest, 55100 Lucca, Italy; 6 Radiotherapy Unit Prato, Presidio Villa Fiorita, Azienda USL Centro Toscana, 59100 Prato, Italy; beatrice.detti@uslcentro.toscana.it; 7 Radiation Oncology Section, Department of Medicine and Surgery, University of Perugia and Perugia General Hospital, 06132 Perugia, Italy

**Keywords:** prostate cancer, genomic classifier, transcriptomic, deep learning, liquid biopsy, PSA, next-generation imaging

## Abstract

High-risk prostate cancer (PC) accounts for 50–75% of 10-year relapse after primary treatment. Routine clinicopathological parameters for PC patient stratification have proven insufficient to inform clinical decisions in this setting. Tumor genomic profiling allowed overcoming the limits of diagnostic accuracy in the field of PC, integrated with radiomic features, automated platforms, evaluation of patient-related factors (age, performance status, comorbidity) and tumor-related factors (risk class, volume, T stage). In this scenario, the use of biomarkers to guide decision-making in localized, high-risk PC is evolving actively and rapidly. Additional tests for prostate-specific antigen have demonstrated superior sensitivity and specificity for detecting clinically significant PC, as well as commercially available genomic classifiers improving the risk prediction of disease recurrence/progression/metastasis, in combination with common clinical variables. This narrative review aimed to summarize the state of the art on the utility and evolution of old and emerging biomarkers in the diagnosis and prognosis of localized, high-risk PC, and the potential for their application in clinical practice. We focused on the theoretical molecular foundation of prostate carcinogenesis and explored the impact of genomic profiling, next-generation sequencing, and artificial intelligence in the extrapolation of customized features able to predict disease aggressiveness and possibly drive personalized therapeutic decisions.

## 1. Introduction

Prostate cancer (PC) is the second most frequent tumor among men worldwide, with an estimated incidence of nearly 1.4 million new cases in 2020, and 7.3% of all newly diagnosed cancers globally in 2022 [1,2]. Localized high-risk PC still accounts for 50–75% of relapse after local primary treatment with curative intent, despite recent advances in diagnosis, and surgical and radiation planning and delivery techniques, owing to increasingly precise and effective treatments [3]. Current guidelines have encoded fairly precise definitions of high-risk PC, based on clinical and pathological staging parameters. D’Amico risk classification is widely adopted by the American Urological Association (AUA), the European Association of Urology (EAU), and the National Institute for Health and Care Excellence (NICE). The presence of clinical T stage ≥ T2c (TNM Staging System, 8th Ed. [4]), Gleason score (GS) ≥ 8, and prostate-specific antigen (PSA) > 20 ng/mL frames this case as high-risk PC. T-stage definition is recommended to be defined on digital rectal examination (DRE), while prostate multiparametric magnetic resonance imaging (mpMRI) can help identify extraprostatic extension of disease, seminal vesicles invasion, and lymph node involvement [5,6,7,8]. The National Comprehensive Cancer Network (NCCN) classification considers high-risk parameters the evidence of extraprostatic extension (clinical T-stage T3a), Gleason score ≥ 8, and PSA ≥ 20 ng/mL, with locally advanced disease (stage cT3b-T4) described as very high-risk PC, instead [9]. Of note, the Radiation Therapy Oncology Group (RTOG) definition considers as high-risk PC any T-stage plus Gleason score ≥ 7 and PSA 20–100 ng/mL, or a clinical T-stage ≥ T2c associated with Gleason score 8–10 and PSA <100 ng/mL [10]. The high rate of clinically relevant treatment failures may be attributable to some main issues. First, aggressive forms of PC drive a high risk of occult micrometastatization (regional and extra-regional lymph nodes, but also distant metastases), which is managed with higher accuracy than in the past thanks to the development of functional imaging with highly prostate tumor-specific radiotracers, namely prostate-specific membrane antigen (PSMA)–positron emission tomography (PET)/computed tomography (CT) [11]. Second, the presence of unfavorable histology patterns. The glomeruloid architecture (the most favorable one) and cribriform, poorly formed, fused patterns are considered different aspects of the Gleason pattern depending on the differentiation grade of the prostatic glandular structure. Attention must be particularly paid to the intermediate risk PC (that is, the International Society of Urological Pathology (ISUP) Grade 2 and 3), as they may unfavorably change the disease prognosis. In particular, the cribriform pattern has been shown to be an independent predictor of recurrence, while the Gleason pattern 4 alone is not [12]. This means that a histologically confirmed PC, Gleason score 3 + 3 with significant cribriform histology, might be at a higher grade and higher risk than a Gleason score 3 + 4 with a less represented cribriform pattern. Additionally, the bi-allelic loss of breast cancer gene 2 (BRCA2) has been associated with a greater extension of the invasive cribriform and intraductal patterns within prostate biopsy specimens, and confirms that the cribriform pattern is suggestive of high-grade PC [13]. Not less important, prostate cancer susceptibility is mainly influenced by aberrations in the homologous recombination repair (HRR) genes (DNA damage repair genes BRCA1/2, DNA damage response gene ataxia-telangiectasia mutated (ATM), partner and localizer BRCA2 (PALB2) and RAD51) and mismatch repair (MMR) genes (MutL protein homolog (MLH)1, MSH2, MSH6, postmeiotic segregation increased 2 (PMS2)). Germline BRCA2 gene mutations have been associated with a higher risk of PC, increased mortality, and earlier age of diagnosis. BRCA1 mutations also increase the risk of PC, although to a lesser extent. Alterations in the ATM, phosphatase and tensin homolog (PTEN), and MYC proto-oncogene have predictive power for the existence of high-grade disease, including occult oligometastases at the time of radical prostatectomy (RP) [14]. Alterations in the DNA repair through phosphorylation of gene checkpoint kinase 2 (CHEK2) or tumor suppressor genes like homeobox B13 (HOXB13) may also occur [15].

**Figure 1:**
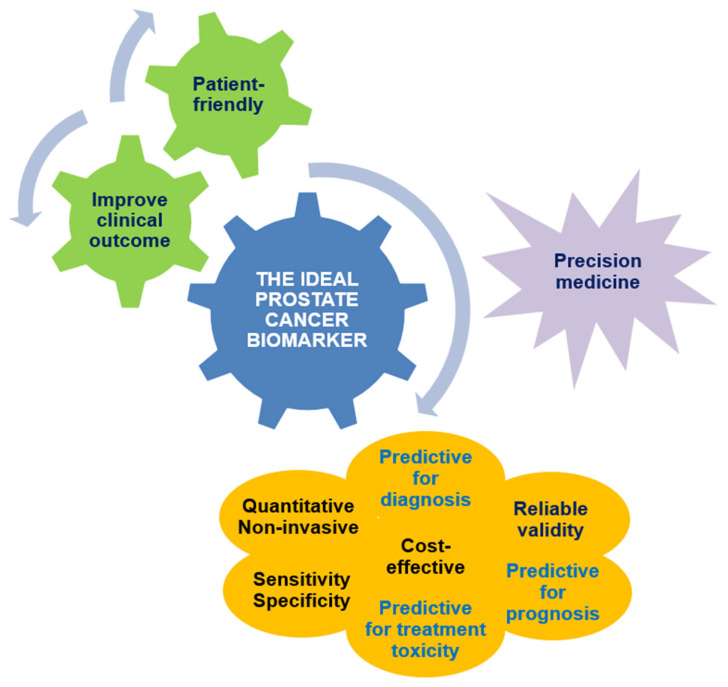
The ideal biomarker characteristics for clinical use in prostate cancer.

The identification of tumor evolution biomarkers to improve the delivery of precision cancer medicine represents the true unmet need. There is an area of active research in the attempt to find blood, urinary, and/or tissue biomarkers able to provide information on the presence or absence of disease, and predict early metastasis following primary treatment, prostate cancer-specific mortality, or the likelihood of treatment response. Which patients with localized PC are more likely to benefit from RP vs. radiotherapy (RT) is of importance in uro-oncology practice, and in assessing if androgen deprivation should be added to RT and for how long; hence, treatment selection should be driven by reliable and unequivocal identification of PC patients at high risk (Figure 1).

We propose a narrative review aimed at providing an overview and update on the current status about the utility and evolution of old and novel biomarkers in the diagnostication and prognostication of localized PC, even in light of the advent of modern genomics and transcriptomics, and increasing convergence of automation and precision medicine.

## 2. Materials and Methods

A comprehensive, non-structured literature research was performed, including Medline, EMBASE, Google Scholar, Scopus, and the Cochrane Library. New evidence was identified, collected, and screened for relevance, covering the emerging issue of molecular biomarkers in the diagnosis and prognosis of localized, high-risk prostate cancer. The search strategy was limited to English-language publications, with a time frame of up to December 2024. We adopted the definition of high-risk PC according to the D’Amico risk classification as the most appropriate and widely shared among the uro-oncology working groups, for the purposes of the review [5,6,7,8]. Selected key words were applied such as “localized prostate cancer”, “high-risk prostate cancer”, “prostate cancer prognostic group”, “genomic hallmarks of prostate cancer”, “genomic heterogeneity in prostate cancer”, “phenotypic heterogeneity in prostate cancer”, “germline mutations in prostate cancer”, “radical prostatectomy”, “radiotherapy and prostate cancer”, “prostate biopsy”, “clinically significant prostate cancer”, “radiation therapy and prostate cancer”, “androgen deprivation signaling pathway”, “prostate-specific antigen for clinically significant prostate cancer”, “tumor markers in prostate cancer”, “liquid biopsy in prostate cancer”, “circulating free DNA in prostate cancer”, “circulating tumor DNA in prostate cancer”, “exosomal RNA in prostate cancer”, “artificial intelligence in localized prostate cancer”, and “deep learning in prostate cancer”.

We thought it appropriate to explore the complex landscape of genomic and phenotypic tumor heterogeneity and downstream molecular profiling in prostate cancer as the common thread for innovation, the object of our research project. We started from the available knowledge on prostate tumor biology and genomic and transcriptomic advances in the field of PC. Then, we stratified the narrative review into three distinct research areas to describe in detail evidence and perspectives on novel biomarkers, and their potential use for diagnostic, prognostic, and predictive purposes, in patients with primary or recurrent, localized, high-risk PC. The role of liquid biopsy and artificial intelligence was also evaluated, as an active and very promising field of research in prostate cancer screening, diagnosis, treatment, and follow up. Finally, we reviewed information and recommendations on such novel PC biomarkers and their possible integration into care decisions within the main national and international guidelines.

## 3. Biomolecular Pitfalls of Prostate Tumorigenesis

Although most men present with localized, potentially curable prostate cancer at onset, current clinical prognostic factors explain only a fraction of the heterogeneity of treatment response. Therefore, these factors do not optimally triage individual patients into a reliable risk grouping that can be used to determine how aggressively the cancer should be treated. Localized prostate cancers exhibit striking inter-tumoral heterogeneity at both genomic and microenvironmental levels. Several reports have documented that more than 80% of primary PCs exhibit multiple topographically and histomorphologically distinct tumor foci. Sequencing efforts have demonstrated a high level of genomic diversity between different patients (inter-patient heterogeneity), but also within a given primary tumor mass (intra-tumoral heterogeneity), as well as its distinct tumor foci and different metastatic sites (inter-tumoral heterogeneity) [16]. Genomic and phenotypic heterogeneity, and microenvironmental influence like selective pressure of cancer treatment driving tumor escape, complicate the assessment of molecular alterations and pose potential barriers to the implementation of precision medicine [16]. Nonetheless, epigenetic mechanisms such as the reduction in mRNA abundance potentially trigger the inactivation of tumor suppression, or DNA methylation patterns may be unique for every lesion with distinct genomic alterations and influence dynamics and relationships among cell clones [16]. In this regard, intermediate-risk PC usually presents as a localized, non-indolent, and clinically heterogeneous disease [17,18]. Despite management with surgery or radiotherapy, about 30% of men suffer relapses; in 10% of cases, rapid biochemical recurrence can portend prostate cancer-specific death. The high metastatic risk 15 to 20 years after diagnosis is the reason why not all the ISUP grade 2 are good candidates for active surveillance (AS) or watchful waiting. Moreover, ISUP 2 has shown a higher risk for developing into high-grade or unfavorable histology, except for Gleason pattern 4 being <5% in less than 50% of core biopsies [17,18]. There is also evidence that a Gleason pattern 3 contiguous to a Gleason pattern 4 area can be molecularly different from a tumor focus Gleason score of 3 + 3 = 6 [16]. A complex relationship among different lesions in primary tumors and metastases has been recently demonstrated, and provided evidence for independent clonal evolution of tumor cell subpopulations [19]. A prostate tumor harbors multiple topographically separated tumor foci, each one showing a distinct, unique, non-overlapping mutation profile and potentially following distinct evolutionary trajectories. Such major biological differences may contribute differently to disease progression and clinical outcomes. The subclonal diversification of prostate tumor foci is directly associated with the aggressiveness of disease: patients with only a single subclone in the index tumor lesion have remarkably good outcomes, while genetically diverse tumors with spatial segregation of distinct cancer cell populations are more likely to develop an increased adaptability to local or systemic treatment [19]. Low-risk monoclonal patients are highly unlikely to experience relapse after definitive local therapy (or, if intermediate-risk, might be evaluated for AS), while polyclonal patients can be further divided based on genomic criteria into good and poor prognosis groups, as aggressive polyclonal tumors are characterized by elevated genomic instability and specific mutational profiles.

Early prostate cancer is largely characterized by the accumulation of single nucleotide variants (SNVs) in genes like forkhead box protein A1 (FOXA1) and ATM, along with deletion of tumor suppressor genes like NKX3-1 and retinoblastoma transcriptional corepressor 1 (RB1). The fusion of the transmembrane protease serine 2 gene with the erythroblast transformation-specific-related gene (TMPRSS2:ERG) on chromosome 21 is the most recurrent genomic alteration in aggressive localized PC, as well as ERG translocations (commons in the peripheral zone, but rare in the transitional zone of the prostate gland) and PTEN loss. Over time, additional driver SNVs accumulate in some tumor cell clones, including the speckle-type POZ protein (SPOP) and TP53. Subclonal driver amplifications in signal transduction pathways like PIK3CA-AKT-mTOR may also occur at this stage [14,19]. When the HRR gene deficiency becomes predominant, the risk of aggressive evolution (biochemical recurrence after local therapy intervention, metastatization) of PC increases even in early localized disease. large numbers of high-risk cell subclones can survive primary, curative treatment in both the prostate gland or occult micrometastatic sites, and hence harbor a network of related locally or systemically expanding subclones [14]. The identification of prostate cancer multiclonality and multifocality is possible when evaluating post-prostatectomy specimens, in theory, while it is more difficult to assess the full tumor micro- and macroheterogeneity on core biopsies. Therefore, systematic prostate mapping may be insufficient for the detection of all the relevant tumor foci. Such findings have important implications for clinical practice in which primary tumor samples are often used to make decisions about actionable alterations in distant metastases [14,16,19]. To overcome this, prostate mpMRI can detect clinically significant, high-risk prostate tumor foci and may conjugate in vivo imaging with morphological, functional, and molecular data to address tumor vulnerability [20,21].

## 4. Genomic Profiling in Localized Prostate Cancer

With the advent of modern next-generation sequencing (NGS) techniques, genomic profiling assays have been brought to the fore in the attempt to better understand insights within the molecular heterogeneity of primary prostate cancer, with the advantage of a relatively low invasive and easily manageable sample collection, like blood or urinary tests [22]. Several bioinformatic analyses have been proposed to identify features predictive of aggressive, high-risk PC or prognostic for survival after primary treatment. In recent years, the most significantly affected biological processes in PC samples have been retrospectively evaluated to look for new potential diagnostic or therapeutic targets. Particularly, the ERG (ETS-mutated gene) rearrangement, as well as PTEN loss, are known to be related to aggressive forms of PC, with higher ISUP grade, locally advanced stage, tumor perineural infiltration, and often lower response rate and poor prognosis after surgery [23,24,25]. Some genes and receptors whose aberrations have never been typically detected in PC patients have also been evaluated. For instance, the human epidermal growth factor receptor 2 (HER2) overexpression has been found with high prevalence in some cohorts of Black men and seems to be related to poor outcomes and rapid growth of prostate tumors, although the role of this oncogene in prostate carcinogenesis remains unclear and controversial [26]. The contribution of the tumor immune response is a further subject of study, despite PC has been extensively described as an immunologically cold tumor, but yet a complex tumor microenvironment (TME) has been shown to surround PC cells, including inflammatory and immune effectors whose role remains poorly understood in this tumor type [27,28,29]. There is a growing interest mainly towards the transcriptome: RNA gene signatures underlying the synthesis of specific proteins and/or the activation of certain signal transduction patterns in response to certain stimuli, such as TME modifications, PC proliferation, invasion and migration, epithelial-to-mesenchymal transition, and therapeutic interventions (irradiation, androgen deprivation, systemic treatment) [30].

## 5. Diagnostic Biomarkers in Localized Prostate Cancer: The Post-PSA Era

To date, blood PSA is the only validated and routine-recommended biomarker for prostate cancer screening and evaluation of treatment response. Screening for PC is a controversial topic in uro-oncology, given the high risk of identifying clinically non-significant cancer and overtreatment while preventing disease-specific mortality. The issues about PSA-based screening are mostly due to its low specificity (nearly 25%) and sensitivity (85%) for cancer and its inability to discriminate between indolent versus significant PC (namely ISUP ≥ 2 and GS ≥ 7) [7,31,32]. Guidelines recommend that the prostate biopsy decision should include factors other than serum PSA level, such as DRE findings, any comorbidities (along with their risk factors, including increasing age and ethnicity), any history of a previous negative biopsy, and, when appropriate, prostate mpMRI features [6,7,9]. At the beginning of the 1990s, different strategies were explored to increase PSA specificity, including PSA density (PSA-D) (ratio of PSA to prostate volume). It has been described as an indicator of reduced risk of aggressive disease for doubtful MRI (i.e., Prostate Imaging Reporting & Data System (PI-RADS) 3), or (PSA-D < 0.15 ng/mL) suggestive for a lower likelihood of having clinically significant prostate cancer (csPC) even in case of PI-RADS 3 [33,34,35,36,37,38]. PSA velocity (change of PSA over a time period) is a controversial indicator of neoplasm aggressiveness, as well. Some modifications to the total PSA (tPSA) assay have also been used, such as the percent free PSA (%fPSA) and the complexed PSA (cPSA), but all with little additional clinical value [39].

Additional tests of PSA have been proposed, showing superior sensitivity and specificity in detecting clinically significant prostate tumors, with a potential utility in avoiding unnecessary biopsies and reducing both overtreatment and the rate of unrecognized high-grade PCs. The position of international guidelines towards these new diagnostic biomarkers is still cautious, since the available evidence is still insufficient to express specific recommendations for their application in clinical practice. Their general indication is that such additional tools may be considered when defining with better accuracy the probability of PC before the initial biopsy or following a first negative biopsy is desirable [6,7,9]. An ongoing clinical trial by the Early Detection Research Network (ClinicalTrials.gov identifier: NCT03784924) is exploring the combination of blood and urinary screening tests with pre-biopsy MRI to assess the optimal sequence of these tools for detecting the majority of csPC, while minimizing patient morbidity and overall costs. Below is a comprehensive summary of the markers identified for potential diagnostic and screening purposes in prostate cancer (Table 1).

_Four-kallikrein score (4K score^®^, OPKO Lab, Nashville, TN, USA): a statistical prediction model combining four serum kallikreins (including tPSA, free PSA (fPSA), initial PSA, and a glycoprotein strongly related with PSA) with clinical data. It was first validated in the Goteborg and Rotterdam arms of the European Randomized Study of Screening for Prostate Cancer (ERSPC), then in a large representative cohort from Sweden. It showed improved prediction of csPC in men with PSA 2–10 ng/mL. The test is currently available only in the United States (US), useful as a reflex test to reduce the number of unnecessary biopsies, or in men with a supposed high risk of csPC, but a previous negative biopsy [39,40,41,42,43,44].

_[-2]proPSA and Prostate Health Index (PHI; Beckman Coulter Inc., Brea, CA, USA): a combination of blood tPSA and isoforms of fPSA deriving from the incomplete removal of a peptide chain from the precursor molecule of the PSA, in 2012 received Food and Drug Administration (FDA) approval for the discrimination of PC versus prostate benign conditions. There are some uncertainties in its true diagnostic accuracy, disagreement on the best PHI cut-off despite 90% of sensitivity preserved, and low level of evidence about its real utility in clinical practice, although it has been shown to reduce the number of unnecessary biopsies and allow a better selection of patients for active surveillance (AS) or more aggressive, curative treatment. The combination of PHI with prostate mpMRI features (PHI density, obtained as the ratio between PHI and the prostate volume in mL) significantly improved sensitivity (up to 100% for PIRADS ≥ 3) for the detection of clinically significant prostate tumors [39,45,46,47,48,49].

_Stockholm-3 test (A3P Biomedical, Stockholm, Sweden): available for clinical use in Sweden, Denmark, Norway, and Finland. It is a predictive model based on clinical variables, serum protein levels, and a genetic score derived from 254 single-nucleotide polymorphisms (SNPs) and an explicit HOXB13 SNP variant [39]. Performed in men with screening PSA level ≥ 4 ng/mL, initially developed in a Swedish population [64], then prospectively validated in a large European population outside Scandinavia [50], and finally in a prospective, multicenter, multiethnic cohort (SEPTA), confirming the test performance in all the racial and ethnic groups (42–52% reduction of benign and ISUP 1 biopsies) [51,52]. The combination of such an algorithm with systematic and mpMRI-targeted biopsy procedures (STHLM3-MRI) resulted in reduced prostate cancer mortality and a contemporary decrease in overdiagnosis (by 69%), biopsy rates (by 8%), and the number of MRI procedures itself (by 36%), compared to a traditional screening strategy (PSA plus prostate mapping biopsy). Since systematic biopsies were performed in men with negative MRI but a very high risk of PC, the detection of low-grade cancer without missing high-risk, clinically significant situations was reduced. Thresholds of ≥11 and ≥15 resulted equally advantageous, so this choice may depend on the physician’s risk preference [53,54].

_Proclarix^®^ (Blue Water Biotech, Inc., Cincinnati, OH, USA): a blood test estimating PC risk based on patient age, tPSA, fPSA, Thrombospondin 1, and Cathepsin D. It performed better than PSA-density and simple ERSPC MRI predictive model in the context of PIRADS 3, and was able to reach 100% detection of csPC, reducing non-significant PC overdiagnosis up to 17% [55].

_Progensa^®^ (Hologic Gen-Probe, Marlborough, MA, USA): based on the urinary identification of non-coding mRNA sequences hyperexpressed in PC (Prostate cancer antigen 3 (PCA 3)). It is mainly recommended to decide whether to repeat biopsy after a first negative procedure, given the reported positive predictive value (PPV) of 80% for PCA 3 score ≥ 60 (sensitivity 42%, specificity 91%), while negative predictive value (NPV) of 88% for PCA3 ≤ 20 (sensitivity 75%, specificity 52%) [56]. The Mi Prostate Score (MiPS) combines tPSA with urinary expression of PCA3 and the molecular signature TMPRSS2:ERG fusion (one of the most represented gene fusions in aggressive PC). It is still considered investigational [39,57].

_SelectMDx^®^ (MDxHealth, Irvine, CA, USA): an algorithm based on urinary mRNA levels of HOXC6, distal-less homeobox 1 (DLX1), and KLK3 (encoding for PSA) genes, combined with age, PSA-density, DRE, and family history of PC. It may be useful in reducing unnecessary biopsy procedures, but harbors a non-negligible percentage of unrecognized clinically significant PC (6.5% of PIRADS < 4 and 3.2% of PIRADS < 3 lesions). Combining this tool with mpMRI increased NPV up to 93%, but the true value of this tool in clinical practice is still unclear and is considered under investigation [39,58,59,60].

_ExoDxTM (MDxHealth, Irvine, CA, USA): based on urinary detection of exosomal RNA from PCA 3, TMPRSS2:ERG, and SPDEF genes [39]. Exosomes originate from cytoplasmic extroflessions, whose cellular secretion has a paracrine or endocrine function of cell communication mediators. Such little extracellular vesicles act as information carriers (i.e., nucleic acids) through specific signaling pathways and adhesion molecules and have been described as responsible for angiogenic signals, modulation of anticancer immune response (especially relations between cells and TME), and cell migration and invasion; thus, they might contribute to signals of (chemo-)radioresistance and/or tumor transformation from localized to systemic [61]. In the field of prostate cancer, this test was born to predict high-grade PC at the initial biopsy. A risk score (range 0–100) of 15.6 is associated with an increased probability of csPC [39,62,63].

_ERSPC Risk Calculators: available online, based on the Dutch arm of the ERSPC trial, it consists of various combinations of clinical variables (i.e., family history, age, urinary symptoms), DRE, tPSA, prostate volume, mpMRI, gene features (PHI), and previous negative biopsy. They have been validated in both European and non-European populations, according to the European guidelines, and may help assess the risk of having PC or indolent PC [7].

_ConfirmMDx^®^ (MDxHealth, Irvine, CA, USA): this is an epigenetic assay, measuring the DNA methylation status of three genes—glutathione-S-transferase P1 (GSTP1), adenomatous polyposis coli (APC), and Ras association domain-containing protein 1 (RASSF1)—in prostate biopsy tissue without cancer [39]. It has the potential to overcome the challenge of negative biopsy in patients with a high probability of clinically significant PC, but is still not FDA approved. Still, NCCN and European guidelines consider it as an option for repeating the biopsy decision after a previous negative one [7,9].

Beyond the Stockholm-3, some of these new screening tools have been tested among Hispanic, Black, and Asian PC patients, with non-unique results and often a low representation of minorities within the recruited cohorts [51].

## 6. Prognostic Biomarkers in Localized Prostate Cancer

Some commercially available molecular biomarker tests have further improved the risk stratification of patients with localized PC. Validated on large, but retrospective cohorts, they showed a possible role in the decision-making, although the long-term influence of such tools in terms of quality of life (QoL), survival, and need for active treatment has not yet been clearly demonstrated. Further studies are required to fully understand how to best incorporate genomic and/or protein-based classifiers in standard clinical practice.

The 2020 American Society of Clinical Oncology (ASCO) recommendations, based on a systematic review by an ASCO multidisciplinary expert panel suggested offering such predictive models to prostate cancer patients whose management was likely to be reliably affected by the combination of a clinically actionable assay result and routine clinical factors. The routine use of molecular biomarkers or the use of additional molecular biomarkers other than commercially available ones is not recommended. The expert panel considered the quality of the available evidence to be insufficient (that is, intermediate) and not supporting the financial and oncologic costs of testing and subsequent disease management [65]. Contemporary, the NCCN guidelines expanded the cohort of patients who can access such molecular algorithms to all men with a life expectancy of at least 10 years and unfavorable intermediate- and high-risk PC [66]. However, no comparative trials among the available assays are available in the literature. Very few studies directly compared genomic scores to MRI features extraction: each one provided clinically relevant information, and there were PC patients for whom combining both information may improve outcomes [67,68,69]. Moreover, evidence about their use in clinical practice lacks robust prospective validation, particularly in the post-prostatectomy setting. The European guidelines still recommend caution in the application of such genomic predictive models in routine clinical practice [7], while some national guidelines (for instance, the Italian ones) do not mention this issue at all [70].

In recent years, the following four biomarker assays have been readily incorporated in multiple retrospective studies to overcome the relatively low accuracy of routine clinicopathological variables in discrimination and prognostication of aggressive, high-risk forms of PC after local primary treatment (Table 2).

_Decipher GC (Decipher Biosciences, San Diego, CA, USA): This is the most extensively studied predictive model among high-risk PC patients [71]. It consists of a 22-gene panel whose tumor tissue corresponds to coding and non-coding RNA expression that occurs in aggressive PC forms [72]. It is scored from 0 to 1, with an established cut-off of 0.45 and 0.60 for low-, intermediate-, and high-GC risk disease classification. Such a genomic risk classifier has been initially validated in the post-prostatectomy setting. Combined with routine clinical and pathological variables, it was able to predict the 10-year PC specific mortality and risk for metastasis with high accuracy in men with adverse pathologic features (pT3, pN1, R1, GS ≥ 7 (4 + 3), early biochemical relapse (BCR) less than 2 years post-surgery, early metastatization after radical prostatectomy (RP), persistently detectable PSA post-RP). It may potentially distinguish patients with biologically indolent PC (i.e., amenable to reduce the duration of androgen deprivation therapy (ADT), when prescribed, but not sufficient to guide postoperative radiotherapy (RT) omission in case of post-prostatectomy PSA persistence even after low-to-intermediate GC risk reclassification) from aggressive disease deserving treatment intensification [73,74]. There has also been an attempt to retrospectively evaluate, but not prospectively test, Decipher GC’s ability to predict the benefit of postoperative radiation [75]. Another retrospective test in a cohort of patients with intermediate-risk PC undergoing radical external beam RT (EBRT) with conventional fractionation without ADT proved GC to outperform other prognostic indices like Risk Class and unfavorable pathologic risk factors in predicting biochemical failure and the occurrence of lethal metastases after RT [76]. Such a biomarker assay has also been validated in pretreatment biopsy specimens from patients enrolled in three NRG/Radiation Therapy Oncology Group (RTOG) 9202, 9413, and 9902 phase III randomized clinical trials, thus treated with EBRT with or without whole pelvis irradiation plus ADT ± systemic treatment intensification. It resulted an independent prognostic factor for distant metastases, death from prostate cancer, and overall survival (OS), and adding Level 1 evidence to the ASCO 2020 guideline [77]. A correlative analysis of the STREAM study (ClinicalTrials.gov identifier: NCT02057939) demonstrated the benefit of intensified ADT (through the addition of 6-month enzalutamide) and concomitant salvage RT in patients with very high-risk and/or node-positive PC after prostatectomy, and identified some transcriptomic signatures available in the Decipher platform. Specifically, PTEN loss (known to be related to the androgen receptor (AR)-blockade resistance) or HRR deficiency, to be associated with worse outcomes (i.e., more rapid relapse, thus more likely to benefit from alternative strategies to ADT, such as docetaxel). Some others, like ADT score, are suggestive for improved outcomes after the addition of second-generation hormonal drugs [78]. There is also an ongoing large, prospective randomized, phase 3 trial, PREDICT-RT (NRG-GU009, NCT04513717), recruiting patients with low Decipher GC risk scores to receive EBRT plus 24-month ADT versus RT plus 12-month ADT, while men with high GC scores or node-positive disease RT plus 24-month ADT with or without intensification with abiraterone and apalutamide. The NRG-GU002 RADD randomized trial (NCT03070886) has been investigating the role of adding adjuvant docetaxel to radiation and androgen blockade in patients with PSA persistence after prostatectomy, instead. Nevertheless, the ECOG ERADICATE trial randomly assigned post-prostatectomy patients with high GC scores to 12 ADT with or without 12 months of darolutamide [79].

_OncotypeDx^®^(GPS™, MDxHealth, Irvine, CA, USA): a prediction model based on differential RNA expression of 17 relevant genes involved in prostate tumor progression. The obtained Genomic Prostate Score (GPS) has proven to predict high-stage and high-grade disease after RP. Additionally, it can accurately reclassify patients with low-risk PC to avoid overtreatment and adequately inform treatment decisions (21% reduction in interventional treatment in favor of AS for low- and very low-risk disease, 35.7% reclassification of low-risk patients into intermediate-risk) [39]. It has been retrospectively evaluated in some cohorts of post-surgical PC patients. It revealed to be an independent predictor of time to BCR regardless of the risk class, in patients with unfavorable intermediate-, high- and very high-risk PC (GPS 41–100), thus more likely to benefit from a multimodal treatment approach along with long-term or intensified ADT, than those with GPS 0–40 who may be evaluated for treatment de-intensification (single treatment modality, or shorter ADT duration) [80,81].

_Prolaris (Myriad Genetics, Inc., Salt Lake City, UT, USA): combines RNA expression analysis of 46 genes involved in cell cycle progression with clinicopathologic information to create a clinical cell-cycle risk (CCR) or cell-cycle progression (CCP) score (range −1.3–4.7) which prognosticates a clinically meaningful different risk of BCR and metastasis for patients with unfavorable intermediate- and high/very high-risk PC submitted to primary RP or RT ± ADT [82]. There is evidence of approximately 27% and 73% of men with high-risk or unfavorable intermediate-risk cancer having CCR scores below the risk threshold (CCR score ≤ 2.112), respectively, so a single local therapeutic strategy instead of a multimodal approach might be considered, as well as concurrent ADT omission in the RT setting [39,83,84,85]. It has also recently been demonstrated to effectively guide AS decisions with durable response over time in a retrospective cohort of intermediate-risk PC patients [86].

_ProMark^®^ (Metamark Genetics, Inc., Cambridge, MA, USA): an immunofluorescence-based assay modeled on the expression of 8 tissue proteins in biopsy samples whose risk score has proven to be prognostic for adverse pathology (GS ≥ 2 and stage ≥ T3b). It demonstrated an 87.2% predictive value for a mildly aggressive tumor with a low D’Amico isk lass score ≤ 0.33, and 76.9% for unfavorable pathology a risk score > 0.8 [87].

Despite the non-negligible amount of promising data, the expensive costs of biomarker assays are not balanced with a clearly demonstrated benefit of sparing potentially unnecessary or ineffective treatment on the QoL of prostate cancer patients, especially when applied to prostate biopsy specimens with their small size and mostly limited cancer volume during PC screening [65,88]. Moreover, the well-described multifocal nature of prostate cancer and related intra- and inter-tumoral biological and molecular heterogeneity, together with prognostically unfavorable histology patterns Gleason 4 satellites (cribriform, intraductal), can make the result of biomarker testing difficult to interpret, even for post-prostatectomy samples. No less important, the potential additional influence of germline DNA mutations (BRCA, ATM, MSH, etc.) on prostate cancer prognostication and decision-making cannot be ignored, including patient preference, patient comorbidities, PSA level, histopathological parameters, and imaging findings (especially in the next-generation imaging (NGI) era). Of note, mathematical models have promisingly suggested a significant correlation between high Decipher scores and risk of upstaging on PSMA-PET/TC, with higher rates of pretreatment evidence of non-localized PC and non-localized or metastatic disease at the functional imaging performed after RP [89,90].

## 7. Predictive Biomarkers in Localized Prostate Cancer: An Open Issue

The above-mentioned biomarker assays have been designed as useful prognostic panels, capable of adequately detecting biologically significant PC, eventually amenable to aggressive treatment intensification. However, none of them has shown any predictive value, which would be necessary to direct individuals to the appropriate anticancer treatment. One predictive biomarker panel, the PAM50 classifier was initially born to guide endocrine-therapy decisions in breast cancer. Then, it has been optimized and retrospectively tested to provide information on the benefit of postoperative ADT and is currently being prospectively evaluated in the randomized NRG GU006 BALANCE trial (NCT03371719), exploring the addition of 6 months of apalutamide to salvage RT in patients with post-prostatectomy BCR.

It has been well established that the BRCA2 mutation addresses worse metastasis-free survival (MFS) and cancer-specific survival (CSS) after primary, radical treatment for localized PC, so whether setting up a pretreatment screening of DNA repair mutations or managing them after the first diagnosis of PC to guide treatment decisions remains a challenge. The idea of a personalized risk prediction may be attractive, but the clinical utility of identifying genomic alterations during the screening for localized PC is still a matter of debate [91]. Unlike the metastatic castration-resistant setting (mCRPC), where carrying HRR mutations gives access to poly-ADP ribose polymerase inhibitor (iPARP) therapy [92,93,94], they do not seem to worsen the risk of cancer progression in newly diagnosed localized disease, provided that an active, primary local treatment with curative intent is proposed [95,96]. Indeed, an estimated 5–15% of patients develop a hereditary form of PC, of whom about 10% harbor HRR or MMR mutations. In addition, somatic mutations (mainly BRCA2 and ATM genes) have been described in 19% of patients with localized PC and 23% of mCRPC, so these findings should not be strictly considered critical for AS exclusion [15,97,98]. Beyond this, germline BRCA1 mutations are associated with an almost 4-fold increased risk of PC, BRCA2 nearly 8.6-fold higher risk than the general population, as well as predisposition to PC onset at an earlier age (less than 55 years) and higher grade, locally advanced or advanced disease, or higher incidence of prostate tumor recurrence after primary treatment. MMR gene mutations also correlate with a higher incidence of PC diagnosis [99,100]. The IMPACT study has been trying to answer the open questions. The protocol aimed to assess the role of PSA screening in men with documented BRCA1, BRCA2, and MMR (MLH1, MLH2, MLH3, MSH2, MSH6, PMS1, PMS2) pathogenic variants. Preliminary data revealed a higher positive predictive value of biopsy in HHR carriers than non-carriers using a PSA cut-off of 3 ng/dl (37.5% vs. 23.3% in BRCA1 alteration and 48% vs. 33.3% of BRCA2-mutated patients, respectively). A subsequent update suggested a higher incidence of PC in MSH2 and MSH6 carriers than in controls [101].

Several SNPs and more than 100 prostate cancer susceptibility loci in low-penetrance genes have been identified in recent years as responsible for nearly 30% of familial relative risk of developing PC. Some of them (rs111906923, rs11568818, rs2735839, rs10993994) are located within or strictly close to genes responsible for cancer predisposition and have been described as potential biomarkers for PC aggressiveness and to predict the risk of metastatization [15]. The PROFILE pilot study (ClinicalTrials.gov identifier: NCT02543905) explored the clinical utility of adding a polygenic risk score modeled on 71 prostate cancer-related SNPs to screening prostate biopsy in men with a family history of PC: 25 out of 100 enrolled patients were diagnosed with PC, 12 (48%) of them with intermediate- to high-risk disease [102]. The incorporation of mpMRI into this prediction algorithm is ongoing.

The protein expressed by the ATM gene has been described to play a crucial role in radiation sensitivity, as it is involved in the detection of DNA double-strand breaks and related cell cycle arrest (to allow the mismatch repair) or apoptosis. In this regard, the SNP named rs1801516 (c.5557G > A, p.Asp1853Asn) has been investigated for increasing the risk of normal tissue radiation toxicity. There was a significant association between this SNP and risk for acute and late toxicity after RT, except for late rectal toxicity in a large meta-analysis including slightly less than 3000 PC patients. Previous smaller studies and meta-analyses have shown a correlation with radiation-induced fibrosis as late complication, likely due to the functional impact of the SNP-induced replacement of the amino acid aspartic acid with the polar asparagine in a functional region of the ATM gene, leading to an increased tendency to cell cycle arrest or apoptosis [103]. At present, there are no suitable SNPs able to predict treatment toxicity with certainty, and/or to be used as prognostic or predictive markers for treatment response. Given advances in the field of next-generation sequencing and bioinformatic analysis, not to mention artificial intelligence (AI), an association of multiple SNPs correlating with a specific endpoint could be reliably hypothesized, with the essential need for prospective validation in large, randomized cohorts.

## 8. Future Perspectives

### 8.1. Liquid Biopsy in Localized Prostate Cancer

Typically, germline testing is conducted using blood, urine, or saliva samples, whereas somatic testing requires tumor or metastatic tissue samples. To date, international recommendations on genetic testing for screening of DNA repair or tumor suppression mutation carriers in newly diagnosed, non-metastatic prostate cancer mainly report low levels of evidence. Of note, any guidelines or consensus statements mention circulating tumor DNA (ctDNA)/cell-free DNA (cfDNA) assays for screening applications in localized PC.

The presence of cfDNA and RNA fragments in human blood was described for the first time in 1948 [104]. They are single- or double-strand fragments of DNA released into peripheral circulation potentially attributable to two main modalities: passive release of nuclear and mitochondrial DNA by apoptotic and necrotic cells, which are usually phagocytosed by the host’s macrophages in physiological conditions; or active, spontaneous release of DNA fragments into the circulatory system by still living cells, as various in-vitro cell cultures have highlighted [105,106]. In cancer patients, a portion of such circulating DNA fragments directly derives from the tumor cells (ctDNA), reproduces their molecular characteristics (SNPs, modifications of the methylation status of regulatory gene sequences, cancer-derived viral sequences) and can be quantified and analyzed for the possible identification of specific tumor-associated genetic aberrations [105,106]. The amount of cfDNA may also be directly correlated with cancer disease burden and with changes in treatment response. In addition, the identification of cancer treatment resistance based on cfDNA genetic alterations seems to anticipate the radiological evidence of the same by 10 months [105].

About prostate cancer, only recently have some clinical studies demonstrated that plasma cfDNA quantification may favor diagnostication and predict biochemical relapse after prostatectomy [107]. Whether this approach paid off in the field of advanced or metastatic PC, especially for mCRPC where elevated cfDNA concentrations have usually been detected providing access to iPARPs target therapy [92,93,94], low or absence of signal from ctDNA, nor somatic allelic mutations have been recorded in the available, little preliminary experiences of plasma sampling from patients with localized PC [108]. To support this, a significant association between cfDNA concentration and clinical characteristics in post-prostatectomy recurrent PC patients was observed by Bastian et al. [109]. Nonetheless, a correlation between shorter average plasma cfDNA fragment size and increased risk of localized PC compared to unhealthy controls has been found, but with low sensitivity and specificity in itself [110]. This means that, at present, the pretreatment evaluation of tumor disease burden in primary PC based on the peripheral identification of tumor-specific somatic alterations is likely to have not a high clinical utility in non-metastatic PC. Prostate tumor heterogeneity further complicates matters, as serial mapping of multiple tumor regions would be necessary to empirically detect mutations shared by almost all or most of the tumor foci and thus also traceable in ctDNA. Moreover, hotspot point mutations in oncogenes and tumor suppressor genes on which commercially available ctDNA assays are focused, such as HRAS and TP53, are rare in PC, even the most recurrent SPOP codon 133 (detected in <5% of newly diagnosed PC). However, a real-world observational experience by Fei and colleagues showed 85.3% of patients with detectable plasma ctDNA before RP developing BCR (compared to only 20.5% of BCR among patients with undetectable pre-treatment ctDNA who experienced longer biochemical progression-free survival (PFS) than the former, anyway), and suggests the possible role of such a biomarker as predictive of unfavorable, more advanced disease [111]. A consistent association between detectable ctDNA and aggressive features of prostate cancer in terms of shorter BCR-free survival and metastasis-free survival (MFS) than patients with undetectable pre-operative ctDNA was also recently reported in a multicentric Australian and United Kingdom (UK) cohort [112].

Overall, circulating tumor cell (CTC) counts may harbor PC micrometastatization, and they are identifiable at an early stage of cancer development. Preliminary translational studies have found pretreatment-positive CTC status (CTC quantification and CTC gene expression) to be potentially predictive for an aggressive form of PC with 93% accuracy, compared to PSA [113,114]. Whether validated in prospective, clinical trials, CTC detection might become a useful and reliable parameter to predict primary local treatment failure in prostate cancer patients [115].

#### 8.1.1. Exosomes and miRNAs

It has been recently hypothesized that exosomes might be involved in building pre-metastatic niches in distant sites from the primary tumor, including draining lymph nodes. In this regard, miRNAs are relatively abundant inside exosomal vesicles (EVs). These are small non-coding RNA molecules of 18–25 nucleotides acting as biological information vectors (proliferation, apoptosis, cell migration, tumor invasion, and angiogenesis signals, therefore able to have great potential for clinical use [61]. A dysregulated, differential expression of urinary miRNA panel and enrichment in some plasma miRNAs between prostate cancer and benign conditions like hyperplasia has also been demonstrated, some of them proportional with the disease severity (pN1, high-risk PC), but only one, urinary EV-derived hsa-miR-126-3p, predictive for PC lymph node invasion in a small, exploratory sample size, with a speculative potential to spare overtreatment and unnecessary complications, such as lymphoedema related to extended pelvic lymphadenectomy, thromboembolism, and lymphatic leakage [116]. There is mounting evidence on the modulation of circulating miRNA expression by ionizing radiation. Among these, miR-141 and miR-106b (known to promote cell proliferation and invasion) resulted differentially expressed in PC culture cell lines of different radiation sensitivity [117]; overexpression of mir-21 has been found in hypoxic conditions and supposed to have a role in radioresistance (well known to mediate AR-induced cell proliferation, apoptosis, epithelial-to-mesenchymal transition) [118]; all of them particularly involved in the DNA repair mechanisms. Namely, low levels of prior-RT miR-106b were suggestive of locally advanced PC, while high levels of miR-21 and miR-106b before and after RT seemed to be correlated with BCR in high-risk patients, and OS [119]. The main limitation of these promising translational data is the lack of standardization and effective screening and validation. Whether such findings would be confirmed on prospective, randomized, large series, net of increasingly sustainable costs, the incorporation of liquid biopsy in the diagnostic routine for primary PC would potentially help guide upfront patient treatment selection (in terms of escalation or de-escalation, benefit from RT, dose and fractionation size, and single or multimodal therapeutic approach) according to the disease aggressiveness, hence to improve long-term benefit and cost-effectiveness of cancer care.

#### 8.1.2. Epigenetic Mechanisms

Post-transcriptional DNA modifications influence gene expression and have a crucial role in carcinogenesis. The extent and location of genomic DNA methylation patterns may predict the presence of cancer and vary according to a specific cancer type. Currently, the tumor methylated fraction can be measured thanks to NGS and has proven to correlate with tumor burden for several cancer types compared with non-cancer individuals [120]. The Circulating Cell-Free Genome Atlas (CCGA) study developed and clinically validated a multicancer early detection test (including urological malignancies) based on peripheral blood detection of the methylation status of cfDNA (ClinicalTrials.gov identifier: NCT02889978), with an 89% accuracy prediction. Such findings were prospectively confirmed with high specificity by the PATHFINDER study (NCT04241796) in an intention-to-treat population [121]. This evolutionary type of detection test is under development for incorporation in commonly used screening programs for patients ≥ 50 years old. About PC, this tool has demonstrated higher sensitivity (prediction accuracy > 90%) in the detection of high-risk, high-grade, and stage IV disease than localized ISUP 1 to 3 forms from the abovementioned two independent clinical trials (sensitivity of 11.2% and 5.6%, respectively), the latter, non-detected cases also showing better OS compared to the same age, stage, and grade Surveillance, Epidemiology, and End Results (SEER) controls [122]. Additionally, hypermethylation of the gene loci Endoglin2 (corresponding protein involved in PC neoangiogenesis, invasion, and migration via transforming growth factor β (TGFβ) signalling) and APC are more frequent in post-prostatectomy specimens of high-risk PC than those with low-risk disease, the latter associated with the additional risk of post-surgical BCR. High levels of DNA methylation had already been described in PC at several gene loci within prostate and periprostatic adipose tissues, mostly in their promoter regions, including glutathione-S-transferase π (GSTP1), Ras association domain family member 1 (RASSF1, unlike benign prostate tissue), death-associated protein kinase 1 (DAPK), runt-related transcription factor 3 (RUNX3), cyclin-dependent kinase inhibitor 2(P14), the tumor necrosis factor receptor superfamily member 10c (TNFRSF10c), and the growth arrest and DNA-damage-inducible α (GADD45) (controlling the apoptotic signaling pathway in PC and responsible for chemosensitivity) [123,124]. Taken together, these data suggest that DNA methylation patterns assessed within ctDNA or prostate tissue might reflect a different level of aggressiveness of PC each time, and create novel insights with a potential diagnostic value, yet difficult to translate into clinical practice.

### 8.2. The Impact of Artificial Intelligence

The development of deep learning algorithms for the analysis of digital images (functional imaging like MRI scans, or digital images mainly of post-surgical histopathology, less frequently of biopsy tissue) is the new frontier of technological advances towards precision medicine in the field of prostate cancer.

Preliminary experiences of AI application to PC prognostication are available in the literature. Certain digital histopathology data, such as Ki67 following hematoxylin-eosin staining or immunohistochemical-based PTEN loss into post-prostatectomy prostate tissue, enters as an input signal and, at the end of the built neural network, is output in the form of risk prediction of disease recurrence, metastasis, or cancer-related death [125,126].

The addition of sophisticated, but affordable automated workflows to stratification of PC patients based on clinicopathological variables, radiomic features, and/or prognostically relevant molecular signatures (PTEN loss, T2:ERG fusion, tissue transcriptomic—mRNA, methylation-based fingerprint), has the potential to reliably affect post-prostatectomy clinical decision. Whether proceed with intensified surveillance or active, escalated or not, adjuvant or salvage treatment especially matters for clinically low-risk patients such as those with negative surgical margins and favorable pathological stage, given the increasing evidence of their ability of identifying patients at higher risk of early BCR and metastasis with high sensitivity and specificity (reported 86%) [125,127,128].

In-training deep learning features in combination with the available risk-stratification nomograms have been promisingly demonstrating to predict the risk of PC aggressiveness more accurately than routine clinicopathological parameters alone also in phase III trials. Such findings, often led to a reclassification of PC patients into a different risk class than conventional assessment, thus may eventually guide more precise, individualized, and fully informed clinical decision making and potentially minimize overtreatment or undertreatment [126,129,130].

## 9. Discussion

The incorporation of biomarker assays into decision-making processes for newly diagnosed, high-risk prostate cancer is evolving actively and rapidly. Current guidelines recommend the use of classical risk classifications, combining clinical, biochemical, and histology data to predict the probability of primary local treatment failure, locoregional and distant metastasis, and death for PC [131,132]. Among these, the EAU risk classification substantially refers to the D’Amico risk classification system, while the Cambridge Prognostic Groups method separates both EAU intermediate- and high-risk disease into clinically relevant subgroups, based on the ISUP grade, T-stage, and PSA levels [7]. The NCCN risk grouping introduces a better stratification of the heterogeneous intermediate-risk disease, with favorable and unfavorable features, like ≥50% of positive biopsy cores and/or the predominance of Gleason pattern 4 [9].

Whether new stratification tools should be preferred over the historical, widely accepted nomograms is still a matter of debate. A 14% risk of reclassification to a higher risk group, and 44% of reclassification from intermediate to a lower risk class than the Cancer of the Prostate Risk Assessment (CAPRA) score has been reported using one of the novel prognostic calculators incorporating imaging, biomarkers, and cognitive or MRI-targeted biopsy information [7,39,133].

PSMA is also highly overexpressed in prostate cancer, especially in undifferentiated, metastatic, and castration-resistant tissues. PSMA-PET/TC is often used for theragnostic selection in the metastatic setting, but whether it can be successfully used for therapeutic prediction and monitoring is still unclear, despite a demonstrated higher specificity than conventional imaging in detecting micrometastases [11,134,135]. Exploratory evidence of the predominance of transcriptomic signatures suggestive of more biologically aggressive disease in PSMA-PET/CT positive for locally advanced or advanced PC has also been provided [90,134,135]. Prostate cancer biomarker use in major international guidelines is summarized in Table 3.

**Figure 2:**
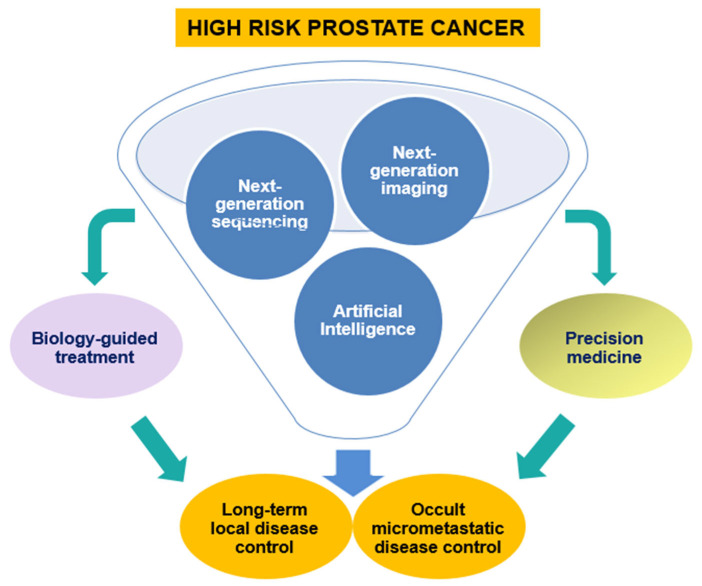
The near future of decision-making for newly diagnosed high-risk prostate cancer.

Together with well-established patient-related issues (age, performance status, comorbidity) and tumor mass-related factors such as tumor size and histological subtype, molecular markers may help to weigh risks against benefits to set up a personalized therapeutic approach aware of the real effectiveness of surveillance/observation or to implement or escalate/de-escalate treatment (Figure 2).

Unfortunately, the available data are insufficient to justify the financial and oncologic costs of routine use of these emerging screening tools, as most of them are still not universally approved or investigational [39,65]. The main limitation of the current application of genomic classifiers in clinical practice relies on the lack of a strong, long-term validation in prospective, randomized, large series that prevents unequivocal conclusions on the true impact on survival and QoL of patients with localized, high-risk PC. Almost all the topics addressed are open fields of research, particularly those targeting inflammation-driven PC or metabolism-driven cancer [140,141]. Emerging translational data still lack standardization in the field of localized PC and cost-effectiveness analyses to definitively assess the influence of molecular biomarkers on decision-making. Despite this, there is an early, but increasingly robust evidence of their strength in defining the probability and aggressiveness of PC with high accuracy, thus reliably informing about the need for active treatment, treatment intensification, or not.

We are aware that the absence of a rigorous, systematic search strategy and a possibly subjective article selection may cause a high risk of bias, difficulty in replicating the research process, and synthesising results. However, the flexibility of a narrative review approach allowed us to provide a complete overview and some key concepts about advances, clinical utility, and limitations of emerging biomarkers in localized, high-grade PC.

The clinical challenges posed by the complex phenotypic and genotypic diversity of prostate cancer foci unavoidably require a multidisciplinary approach. Insights from imaging and molecular pathology studies will need to be paired with careful clinical annotations to establish features of clinically relevant heterogeneity. Soon, novel computational approaches and deep learning algorithms will be desirable to analyze the resulting multidimensional data. The integration of different levels of information, capturing features of tumor vulnerability, will provide an in-depth understanding of tumor biology and will allow us to overcome obstacles to precision medicine due to intra- and inter-tumoral heterogeneity [126,127,128].

Integrated, automated workflows have the potential to serve precision medicine aims to set and implement unbiased, quantitative approaches able to improve disease prognostication and the prediction of treatment response; hence, cancer patients can be stratified for increasingly accurate, well-tolerated, cost-effective, and high-quality cancer care.

## 10. Conclusions

The identification of clinically relevant biomarkers, both current and emerging, is crucial to improve the management of prostate cancer. Provided the unquestionable importance of blood PSA test, the application of molecular markers in clinical practice may ensure more accurate and less invasive diagnosis, offer valuable prognostic information, and enable the development of highly personalized treatment plans. Long-term validation of such novel molecular markers based on blood, urine and/or prostate specimens within large, prospective randomized series is warranted to establish their use as standard of care. The detection of the unique tumor biology for each PC patient in terms of genomic profiling and radiomic and clinical features may potentially improve risk assessment for patients with high-risk PC, and makes it possible to select the most effective treatment options, avoiding ineffective interventions and reducing unnecessary toxicity and overtreatment for low-risk disease.

## Figures and Tables

**Table 1:** Novel markers identified for potential diagnostic and screening purposes in prostate cancer.

Marker	Composition	Sample Type	Description	
*Four-kallikrein score (4K score^®^)*	_tPSA _fPSA _iPSA _human kallikrein 2 combined with clinical data (age, DRE, previous biopsy)	Blood	Available in the US Able to predict high-grade PC and the risk of metastatization	[39,40,41,42,43,44]
*[-2]proPSA and Prostate Health Index (PHI)*	_tPSA _fPSA isoforms from PSA precursor PHI density The ratio between PHI and mpMRI prostate volume	Blood	Available in the US Better diagnostic performance for PSA 2–10 ng/mL than PSA derivatives Uncertain diagnostic accuracy Up to 100% sensitivity for csPC detection with PHI density	[39,45,46,47,48,49]
*Stockholm-3 test*	_age _family history of PC _previous biopsy _tPSA _fPSA/tPSA ratio _human kallikrein 2 _macrophage inhibitory cytokine-1 _microseminoprotein-β [MSMB] _polygenic risk score (SNPs and HOXB13 SNP variant)	Blood	Available in the US and Scandinavian countries Able to predict csPC especially in combination with mpMRI	[39,50,51,52,53,54]
*Proclarix^®^*	_age _tPSA _fPSA _Thrombospondin 1 _Cathepsin D	Blood	Up to 100% detection of csPC especially for PIRADS 3 mpMRI cases	[55]
*Progensa^®^*	Hyperexpressed non-coding mRNA sequences (PCA 3) Investigational: Mi Prostate Score (MiPS) combination of tPSA with PCA 3 and the molecular signature TMPRSS2:ERG fusion	Urine	80% PPV for PCA 3 score ≥ 60 (sensitivity 42%, specificity 91%) 88% NPV for PCA 3 ≤ 20 (sensitivity 75%, specificity 52%)	[39,56,57]
*SelectMDx^®^*	_HOXC6 mRNA _DLX1 mRNA _KLK3 mRNA combined with clinical information (age, PSA density, DRE, and family history of PC)	Urine	Considered under investigation Unrecognized 6.5% of PIRADS < 4 and 3.2% of PIRADS < 3 csPC Up to 93% NPV in combination with mpMRI	[39,58,59,60]
*ExoDxTM*	_PCA 3 exosomal RNA _TMPRSS2:ERG exosomal RNA _SPDEF exosomal RNA	Urine	Still investigational 15.6 Risk score (range 0–100) associated with increased probability of csPC	[39,61,62,63]
*ERSPC Risk Calculators*	_tPSA _prostate volume _mpMRI features _PHI combined with clinical variables (family history of PC, age, urinary symptoms, DRE, previous negative biopsy)	Mixed	Available online Validated in European and non-European populations Helpful in assessing the risk of csPC	[7]
*ConfirmMDx^®^*	_ GSTP1 DNA methylation status _APC DNA methylation status _RASSF1 DNA methylation status	Prostate biopsy	Not FDA approved An option for NCCN and European guidelines Able to overcome the challenge of negative biopsy for high probability of csPC	[7,9]

tPSA = total PSA; fPSA = free PSA; iPSA = initial PSA; DRE = digital rectal examination; US = United States; PC = prostate cancer; mpMRI = multiparametric magnetic resonance imaging; csPC = clinically significant prostate cancer; SNP(s) = single nucleotide polymorphism(s); PCA 3 = prostate cancer antigen 3; PIRADS = Prostate Imaging Reporting and Data System; PPV = positive predictive value; NPV = negative predictive value; ERSPC = European Randomized Study of Screening for Prostate Cancer; FDA = Food and Drug Administration; NCCN = National Comprehensive Cancer Network.

**Table 2:** Commercially available prognostic assays for localized prostate cancer.

Marker	Composition	Sample Type	Description	
*Decipher GC*	22-gene panel expressed in aggressive PC forms	Prostate tissue (post-prostatectomy and biopsy)	0.45 and 0.60 cut-off for risk classification Able to predict post-prostatectomy 10y- PC specific mortality and risk for metastasis in case of adverse pathologic features and guide adjuvant treatment decision Prognostic factor for biochemical failure, lethal distant metastases, death for prostate cancer, and OS after primary RT	[71,72,73,74,75,76,77,78,79]
*OncotypeDx^®^*(*GPS™*)	17 relevant genes for PC progression GPS 0–100	Prostate tissue (post-prostatectomy and biopsy)	Independent predictor of high- and very high-risk PC (GPS 41–100) and time to BCR 21% reduction of interventional treatment for low- and very low-risk disease 35.7% reclassification of low-risk patients into intermediate-risk PC	[39,80,81]
*Prolaris*	46 genes involved in cell cycle progression combined with clinicopathologic information CCR/CCP score −1.3–4.7	Prostate tissue (post-prostatectomy and biopsy)	Risk threshold CCR score ≤ 2.112 prognosticates a clinically meaningful different risk of BCR and metastasis Able to guide AS decision	[39,82,83,84,85,86]
*ProMark^®^*	8 tissue proteins	Prostate biopsy	87.2% predictive value for little aggressive tumor in low-risk PC at a score ≤ 0.33 76.9% predictive value for unfavorable pathology at a risk score > 0.8	[87]

PC = prostate cancer; OS = overall survival; RT = radiotherapy; GPS = genomic prostate score; BCR = biochemical recurrence; CCR = clinical cell-cycle risk; CCP = cell-cycle progression; AS = active surveillance.

**Table 3:** Prostate cancer biomarker use in major international guidelines.

Marker	EAU 2025 [7]	NCCN 2025 [9]	ASCO 2020–2023 [65,136]	AUA 2022–2023 [32,137,138]	APCCC 2023 [139]	NICE 2021 [8]
*Four-kallikrein score (4K score^®^)*	None	None	None	Not clear utility for reducing unnecessary biopsies	Minimize unnecessary prostate biopsies in men tested with PSA	None
*[-2]proPSA and Prostate Health Index (PHI)*	None	Not clear usefulness	None	Improve the detection of clinically significant prostate cancer	Reduce the number of unnecessary prostate biopsies in PSA-tested men	Not recommended
*Stockholm-3 test*	None	Not clear usefulness	None	Not clear utility for reducing unnecessary biopsies	Reduce the percentage of clinically insignificant cancers when used in combination with MRI in a PSA screening population	None
*Proclarix^®^*	None	None	None	Not clear utility for reducing unnecessary biopsies	Correlated with the detection of clinically significant PC, notably in the case of equivocal MRI	None
*Progensa^®^*	None	None	None	Not mentioned	Not clear usefulness	Not recommended
*SelectMDx^®^*	None	None	None	Not clear utility for reducing unnecessary biopsies	Not clear usefulness	None
*ExoDxTM*	None	None	None	Not clear utility for reducing unnecessary biopsies	Investigational	None
*ERSPC Risk Calculators*	None	None	None	Provide estimates that facilitate clinician/patient discussion of detection risk, but may differ for subgroups	Could help in discriminating between aggressive and non-aggressive tumors	None
*ConfirmMDx^®^*	None	None	None	Not clear utility for reducing unnecessary biopsies	Not mentioned	None
*Decipher GC*	Predictor of metastasis after RP, but to be used only in clinical trials	Recommended to inform adjuvant treatment post RP Considered as part of counselling for risk stratification in patients with PSA resistance/relapse after RP (category 2B)	Some clinical data Routine use not recommended in the absence of prospective trials	To be validated in prospective clinical trials Routine use not recommended	Prognostic role Routine use not recommended in the absence of prospective trials	None
*OncotypeDx^®^(GPS™)*	To be used only in clinical trials	Part of counselling for risk stratification	Some clinical data Routine use not recommended in the absence of prospective trials	To be validated in prospective clinical trials Routine use not recommended	Prognostic role Routine use not recommended in the absence of prospective trials	None
*Prolaris*	To be used only in clinical trials	Part of counselling for risk stratification	Some clinical data Routine use not recommended in the absence of prospective trials	To be validated in prospective clinical trials Routine use not recommended	Prognostic role Routine use not recommended in the absence of prospective trials	None
*ProMark^®^*	None	Not mentioned	Some clinical data Routine use not recommended in the absence of prospective trials	Not mentioned	Prognostic role Routine use not recommended in the absence of prospective trials	None
*PAM50*	None	None	None	None	None	None

PSA = prostate-specific antigen; MRI = magnetic resonance imaging; RP = radical prostatectomy; ERSPC = European Randomized Study of Screening for Prostate Cancer; EAU = European Association of Urology; NCCN = National Comprehensive Cancer Network; ASCO = negative predictive value; AUA = American Urological Association; APCCC = Advanced Prostate Cancer Consensus Conference; NICE = National Institute for Health and Care Excellence.

## Data Availability

No new data were created or analyzed in this study, not applicable.

## Abbreviations

The following abbreviations are used in this manuscript:

%fPSA	Percent free PSA
ADT	Androgen deprivation therapy
AI	Artificial intelligence
APC	Adenomatous polyposis coli
AR	Androgen receptor
AS	Active surveillance
ASCO	American Society of Clinical Oncology
ATM	Ataxia-telangiectasia mutated
AUA	American Urological Association
BCR	Biochemical relapse
BRCA	Breast cancer gene
CAPRA	Cancer of the Prostate Risk Assessment
CCP	Cell-cycle progression
CCR	Clinical cell-cycle risk
cfDNA	Cell-free DNA
CHEK2	Checkpoint kinase 2
cPSA	Complexed PSA
csPC	Clinically significant prostate cancer
CSS	Cancer-specific survival
CTCs	Circulating tumor cells
ctDNA	Circulating tumor DNA
DAPK	Death-associated protein kinase 1
DLX1	Distal-less homeobox 1
DRE	Digital rectal examination
EAU	European Association of Urology
EBRT	External beam RT
ERSPC	European Randomised Study for Screening of Prostate Cancer
FDA	Food and Drug Administration
FOXA1	Forkhead box protein A1
fPSA	Free PSA
GADD45	Growth arrest and DNA-damage-inducible α
GPS	Genomic Prostate Score
GS	Gleason score
GSTP1	Glutathione-S-transferase P1 / Glutathione-S-transferase π
HER2	Human epidermal growth factor receptor 2
HOXB13	Omeobox B13
HRR	Homologous recombination repair
iPARP	Poly-ADP ribose polymerase RASSF inhibitor
ISUP	International Society of Urological Pathology
mCRPC	Metastatic castration-resistant prostate cancer
MFS	Metastasis-free survival
MiPS	Mi Prostate Score
MLH1	MutL protein homolog 1
MMR	Mismatch repair
MpMRI	Multiparametric magnetic resonance imaging
NCCN	National Comprehensive Cancer Network
NGI	Next-generation imaging
NICE	National Institute for Health and Care Excellence
NPV	Negative predictive value
OS	Overall survival
PALB2	Partner and localizer of BRCA2
PC	Prostate cancer
PCA 3	Prostate cancer antigen 3
PHI	Prostate Health Index
PI-RADS	Prostate Imaging Reporting & Data System
PMS2	Postmeiotic segregation increased 2
PSA	Prostate-specific antigen
PSA-D	PSA density
PSMA-PET/CT	Prostate-specific membrane antigen–positron emission tomography/computed tomography
PTEN	Phosphatase and tensin homolog
QoL	Quality of life
RASSF1	Ras association domain family member 1 / Ras association domain-containing protein 1
RB1	Retinoblastoma transcriptional corepressor
RP	Radical prostatectomy
RT	Radiotherapy
RTOG	Radiation Therapy Oncology Group
RUNX3	Runt-related transcription factor 3
SEER	Surveillance, Epidemiology, and End Results
SNPs	Single-nucleotide polymorphisms
SNVs	Single-nucleotide variants
SPOP	Speckle-type POZ protein
TGFβ	Transforming growth factor β
TME	Tumor microenvironment
TMPRSS2:ERG	The fusion of the transmembrane protease serine 2 gene with the erythroblast transformation-specific-related gene
TNFRSF10c	Tumor necrosis factor receptor superfamily member 10c
tPSA	Total PSA
US	United States

## References

[1] Culp M.B., Soerjomataram I., Efstathiou J.A., Bray F., Jemal A. (2020). Recent Global Patterns in Prostate Cancer Incidence and Mortality Rates. Eur. Urol..

[2] Bray F., Laversanne M., Sung H., Ferlay J., Siegel R.L., Soerjomataram I., Jemal A. (2024). Global cancer statistics 2022: GLOBOCAN estimates of incidence and mortality worldwide for 36 cancers in 185 countries. CA Cancer J. Clin..

[3] Hussain M., Lin D., Saad F., Vapiwala N., Chapin B.F., Sandler H., Evans C.P., Carducci M.A., Sachdev S. (2021). Newly Diagnosed High-Risk Prostate Cancer in an Era of Rapidly Evolving New Imaging: How Do We Treat?. J. Clin. Oncol..

[4] Brierley J.D., Gospodarowicz M.K., Wittekind C. (2017). TNM Classification of Malignant Tumours.

[5] D’Amico A.V., Whittington R., Malkowicz S.B., Schultz D., Blank K., Broderick G.A., Tomaszewski J.E., Renshaw A.A., Kaplan I., Beard C.J. (1998). Biochemical outcome after radical prostatectomy, external beam radiation therapy, or interstitial radiation therapy for clinically localized prostate cancer. JAMA.

[6] Eastham J.A., Auffenberg G.B., Barocas D.A., Chou R., Crispino T., Davis J.W., Eggener S., Horwitz E.M., Kane C.J., Kirkby E. (2022). Clinically Localized Prostate Cancer: AUA/ASTRO Guideline, Part I: Introduction, Risk Assessment, Staging, and Risk-Based Management. J. Urol..

[7] Cornford P., van den Bergh R.C.N., Briers E., Van den Broeck T., Brunckhorst O., Darraugh J., Eberli D., De Meerleer G., De Santis M., Farolfi A. (2024). EAU-EANM-ESTRO-ESUR-ISUP-SIOG Guidelines on Prostate Cancer-2024 Update. Part I: Screening, Diagnosis, and Local Treatment with Curative Intent. Eur. Urol..

[8] Dasgupta P., Davis J., Hughes S. (2019). NICE guidelines on prostate cancer 2019. BJU Int..

[9] NCCN Clinical Practice Guidelines in Oncology (NCCN Guidelines®) Prostate Cancer Version24.2025—April 16, 2025. https://www.nccn.org/patients/guidelines/content/PDF/prostate-early-patient.pdf.

[10] Roach M., Lu J., Pilepich M.V., Asbell S.O., Mohiuddin M., Terry R., Grignon D. (2000). Four prognostic groups predict long-term survival from prostate cancer following radiotherapy alone on Radiation Therapy Oncology Group clinical trials. Int. J. Radiat. Oncol. Biol. Phys..

[11] Hofman M.S., Lawrentschuk N., Francis R.J., Tang C., Vela I., Thomas P., Rutherford N., Martin J.M., Frydenberg M., Shakher R. (2020). proPSMA Study Group Collaborators. Prostate-specific membrane antigen PET-CT in patients with high-risk prostate cancer before curative-intent surgery or radiotherapy (proPSMA): A prospective, randomized, multicentre study. Lancet.

[12] Downes M.R., Xu B., van der Kwast T.H. (2021). Cribriform architecture prostatic adenocarcinoma in needle biopsies is a strong independent predictor for lymph node metastases in radical prostatectomy. Eur. J. Cancer.

[13] Lozano R., Salles D.C., Sandhu S., Aragón I.M., Thorne H., López-Campos F., Rubio-Briones J., Gutierrez-Pecharroman A.M., Maldonado L., di Domenico T. (2021). Association between BRCA2 alterations and intraductal and cribriform histologies in prostate cancer. Eur. J. Cancer.

[14] Fraser M., Sabelnykova V.Y., Yamaguchi T.N., Heisler L.E., Livingstone J., Huang V., Shiah Y.J., Yousif F., Lin X., Masella A.P. (2017). Genomic hallmarks of localized, non-indolent prostate cancer. Nature.

[15] Marino F., Totaro A., Gandi C., Bientinesi R., Moretto S., Gavi F., Pierconti F., Iacovelli R., Bassi P., Sacco E. (2023). Germline mutations in prostate cancer: A systematic review of the evidence for personalized medicine. Prostate Cancer Prostatic Dis..

[16] Haffner M.C., Zwart W., Roudier M.P., True L.D., Nelson W.G., Epstein J.I., De Marzo A.M., Nelson P.S., Yegnasubramanian S. (2021). Genomic and phenotypic heterogeneity in prostate cancer. Nat. Rev. Urol..

[17] Hamdy F.C., Donovan J.L., Lane J.A., Metcalfe C., Davis M., Turner E.L., Martin R.M., Young G.J., Walsh E.I., Bryant R.J. (2023). ProtecTStudy Group Fifteen-Year Outcomes after Monitoring Surgery or Radiotherapy for Prostate Cancer. N. Engl. J. Med..

[18] Lam T.B.L., MacLennan S., Willemse P.M., Mason M.D., Plass K., Shepherd R., Baanders R., Bangma C.H., Bjartell A., Bossi A. (2019). EAU-EANM-ESTRO-ESUR-SIOG Prostate Cancer Guideline Panel Consensus Statements for Deferred Treatment with Curative Intent for Localised Prostate Cancer from an International Collaborative Study (DETECTIVE Study). Eur. Urol..

[19] Espiritu S.M.G., Liu L.Y., Rubanova Y., Bhandari V., Holgersen E.M., Szyca L.M., Fox N.S., Chua M.L.K., Yamaguchi T.N., Heisler L.E. (2018). The Evolutionary Landscape of Localized Prostate Cancers Drives Clinical Aggression. Cell.

[20] Das C.J., Razik A., Netaji A., Verma S. (2020). Prostate MRI-TRUS fusion biopsy: A review of the state of the art procedure. Abdom. Radiol..

[21] Thorwarth D. (2018). Biologically adapted radiation therapy. Z. Med. Phys..

[22] Cancer Genome Atlas Research Network (2015). The Molecular Taxonomy of Primary Prostate Cancer. Cell.

[23] Brady L., Carlsson J., Baird A.M., Casey O., Vlajnic T., Murchan P., Cormican D., Costigan D., Gray S., Sheils O. (2021). Correlation of integrated ERG/PTEN assessment with biochemical recurrence in prostate cancer. Cancer Treat. Res. Commun..

[24] Leinonen K.A., Saramäki O.R., Furusato B., Kimura T., Takahashi H., Egawa S., Suzuki H., Keiger K., Ho Hahm S., Isaacs W.B. (2013). Loss of PTEN is associated with aggressive behavior in ERG-positive prostate cancer. Cancer Epidemiol. Biomarkers Prev..

[25] Kobelyatskaya A.A., Pudova E.A., Katunina I.V., Snezhkina A.V., Fedorova M.S., Pavlov V.S., Kotelnikova A.O., Nyushko K.M., Alekseev B.Y., Krasnov G.S. (2023). Transcriptome Profiling of Prostate Cancer, Considering Risk Groups and the TMPRSS2-ERG Molecular Subtype. Int. J. Mol. Sci..

[26] Mavingire N., Moore J.C., Johnson J.R., Dwead A.M., Cropp C.D., Mechref Y., Kobeissy F., Rais-Bahrami S., Woods-Burnham L. (2024). Revisiting HER2 in Prostate Cancer from an Inclusive Perspective: From Biomarkers to Omics. Cancers.

[27] Xie B., Li K., Zhang H., Lai G., Li D., Zhong X. (2022). Identification and validation of an immune-related gene pairs signature for three urologic cancers. Aging.

[28] Yanai Y., Kosaka T., Mikami S., Hongo H., Yasumizu Y., Takeda T., Matsumoto K., Miyauchi J., Kitano S., Oya M. (2021). CD8-positive T cells and CD204-positive M2-like macrophages predict postoperative prognosis of very high-risk prostate cancer. Sci. Rep..

[29] Magrowski Ł., Masri O., Ciepał J., Depowska G., Nowicka Z., Stando R., Chimiak K., Bylica G., Czapla B., Masri M. (2022). Pre-Treatment Hemoglobin Concentration and Absolute Monocyte Count as Independent Prognostic Factors for Survival in Localized or Locally Advanced Prostate Cancer Patients Undergoing Radiotherapy. Biomedicines.

[30] Olczak M., Orzechowska M.J., Bednarek A.K., Lipiński M. (2023). The Transcriptomic Profiles of ESR1 and MMP3 Stratify the Risk of Biochemical Recurrence in Primary Prostate Cancer beyond Clinical Features. Int. J. Mol. Sci..

[31] Kang D.W., Barnes O., Vander Heiden M.G., Dieli-Conwright C.M. (2022). Effect of exercise on tumor markers—Is exercise anti-tumorigenic in humans?: A scoping review of preliminary clinical investigations. Crit. Rev. Oncol. Hematol..

[32] Wei J.T., Barocas D., Carlsson S., Coakley F., Eggener S., Etzioni R., Fine S.W., Han M., Kim S.K., Kirkby E. (2023). Early Detection of Prostate Cancer: AUA/SUO Guideline Part I: Prostate Cancer Screening. J. Urol..

[33] Schröder F.H., Roobol M.J., van der Kwast T.H., Kranse R., Bangma C.H. (2006). Does PSA velocity predict prostate cancer in pre-screened populations?. Eur. Urol..

[34] Wolters T., Roobol M.J., Bangma C.H., Schröder F.H. (2009). Is prostate-specific antigen velocity selective for clinically significant prostate cancer in screening? European Randomized Study of Screening for Prostate Cancer (Rotterdam). Eur. Urol..

[35] Stephan C., Stroebel G., Heinau M., Lenz A., Roemer A., Lein M., Schnorr D., Loening S.A., Jung K. (2005). The ratio of prostate-specific antigen (PSA) to prostate volume (PSA density) as a parameter to improve the detection of prostate carcinoma in PSA values in the range of <4 ng/mL. Cancer.

[36] Petrelli F., Vavassori I., Cabiddu M., Coinu A., Ghilardi M., Borgonovo K., Lonati V., Barni S. (2016). Predictive Factors for Reclassification and Relapse in Prostate Cancer Eligible for Active Surveillance: A Systematic Review and Meta-analysis. Urology.

[37] Maggi M., Panebianco V., Mosca A., Salciccia S., Gentilucci A., Di Pierro G., Busetto G.M., Barchetti G., Campa R., Sperduti I. (2020). Prostate Imaging Reporting and Data System 3 Category Cases at Multiparametric Magnetic Resonance for Prostate Cancer: A Systematic Review and Meta-analysis. Eur. Urol. Focus..

[38] Pagniez M.A., Kasivisvanathan V., Puech P., Drumez E., Villers A., Olivier J. (2020). Predictive Factors of Missed Clinically Significant Prostate Cancers in Men with Negative Magnetic Resonance Imaging: A Systematic Review and Meta-Analysis. J. Urol..

[39] Garrido M.M., Bernardino R.M., Marta J.C., Holdenrieder S., Guimarães J.T. (2022). Tumour markers in prostate cancer: The post-prostate-specific antigen era. Ann. Clin. Biochem..

[40] Parekh D.J., Punnen S., Sjoberg D.D., Asroff S.W., Bailen J.L., Cochran J.S., Concepcion R., David R.D., Deck K.B., Dumbadze I. (2015). A multi-institutional prospective trial in the USA confirms that the 4Kscore accurately identifies men with high-grade prostate cancer. Eur. Urol..

[41] Braun K., Sjoberg D.D., Vickers A.J., Lilja H., Bjartell A.S. (2016). A Four-kallikrein Panel Predicts High-grade Cancer on Biopsy: Independent Validation in a Community Cohort. Eur. Urol..

[42] Lin D.W., Newcomb L.F., Brown M.D., Sjoberg D.D., Dong Y., Brooks J.D., Carroll P.R., Cooperberg M., Dash A., Ellis W.J. (2017). Evaluating the Four Kallikrein Panel of the 4Kscore for Prediction of High-grade Prostate Cancer in Men in the Canary Prostate Active Surveillance Study. Eur. Urol..

[43] Olleik G., Kassouf W., Aprikian A., Hu J., Vanhuyse M., Cury F., Peacock S., Bonnevier E., Palenius E., Dragomir A. (2018). Evaluation of New Tests and Interventions for Prostate Cancer Management: A Systematic Review. J. Natl. Compr. Cancer Netw..

[44] Stovsky M., Klein E.A., Chait A., Manickam K., Stephenson A.J., Wagner M., Dineen M., Lotan Y., Partin A., Baniel J. (2019). Clinical Validation of IsoPSA™, a Single Parameter, Structure Based Assay for Improved Detection of High Grade Prostate Cancer. J. Urol..

[45] Catalona W.J., Partin A.W., Sanda M.G., Wei J.T., Klee G.G., Bangma C.H., Slawin K.M., Marks L.S., Loeb S., Broyles D.L. (2011). A multicenter study of [-2]pro-prostate specific antigen combined with prostate specific antigen and free prostate specific antigen for prostate cancer detection in the 2.0 to 10.0 ng/ml prostate specific antigen range. J. Urol..

[46] Guazzoni G., Lazzeri M., Nava L., Lughezzani G., Larcher A., Scattoni V., Gadda G.M., Bini V., Cestari A., Buffi N.M. (2012). Preoperative prostate-specific antigen isoform p2PSA and its derivatives, %p2PSA and prostate health index, predict pathologic outcomes in patients undergoing radical prostatectomy for prostate cancer. Eur. Urol..

[47] Tosoian J.J., Druskin S.C., Andreas D., Mullane P., Chappidi M., Joo S., Ghabili K., Agostino J., Macura K.J., Carter H.B. (2017). Use of the Prostate Health Index for detection of prostate cancer: Results from a large academic practice. Prostate Cancer Prostatic Dis..

[48] Loeb S., Catalona W.J. (2014). The Prostate Health Index: A new test for the detection of prostate cancer. Ther. Adv. Urol..

[49] de la Calle C., Patil D., Wei J.T., Scherr D.S., Sokoll L., Chan D.W., Siddiqui J., Mosquera J.M., Rubin M.A., Sanda M.G. (2015). Multicenter Evaluation of the Prostate Health Index to Detect Aggressive Prostate Cancer in Biopsy Naïve Men. J. Urol..

[50] Elyan A., Saba K., Sigle A., Wetterauer C., Engesser C., Püschel H., Attianese S., Maurer P., Deckart A., Cathomas R. (2024). Prospective Multicenter Validation of the Stockholm3 Test in a Central European Cohort. Eur. Urol. Focus..

[51] Vigneswaran H.T., Eklund M., Discacciati A., Nordström T., Hubbard R.A., Perlis N., Abern M.R., Moreira D.M., Eggener S., Yonover P. (2024). SEPTASTHLM3 Study Group Stockholm3 in a Multiethnic Cohort for Prostate Cancer Detection (SEPTA): AProspective Multicentered Trial. J. Clin. Oncol..

[52] Brooks J.D. (2024). Stockholm3 in a Multiethnic Cohort: Optimizing Prostate Cancer Screening to Reduce Harm and Improve Equity. J. Clin. Oncol..

[53] Nordström T., Discacciati A., Bergman M., Clements M., Aly M., Annerstedt M., Glaessgen A., Carlsson S., Jäderling F., Eklund M. (2021). STHLM3 study group. Prostate cancer screening using a combination of risk-prediction, MRI, and targeted prostate biopsies (STHLM3-MRI): A prospective, population-based, randomised, open-label, non-inferiority trial. Lancet Oncol..

[54] Björnebo L., Discacciati A., Falagario U., Vigneswaran H.T., Jäderling F., Grönberg H., Eklund M., Nordström T., Lantz A. (2024). Biomarker vs MRI-Enhanced Strategies for Prostate Cancer Screening: The STHLM3-MRI Randomized Clinical Trial. JAMA Netw. Open.

[55] Morote J., Campistol M., Triquell M., Celma A., Regis L., de Torres I., Semidey M.E., Mast R., Santamaria A., Planas J. (2022). Improving the Early Detection of Clinically Significant Prostate Cancer in Men in the Challenging Prostate Imaging-Reporting and Data System 3 Category. Eur. Urol. Open Sci..

[56] Tosoian J.J., Zhang Y., Xiao L., Xie C., Samora N.L., Niknafs Y.S., Chopra Z., Siddiqui J., Zheng H., Herron G. (2024). EDRN-PCA3 Study Group. Development and Validation of an 18-Gene Urine Test for High-Grade Prostate Cancer. JAMA Oncol..

[57] Tomlins S.A., Rhodes D.R., Perner S., Dhanasekaran S.M., Mehra R., Sun X.W., Varambally S., Cao X., Tchinda J., Kuefer R. (2005). Recurrent fusion of TMPRSS2 and ETS transcription factor genes in prostate cancer. Science.

[58] Van Neste L., Hendriks R.J., Dijkstra S., Trooskens G., Cornel E.B., Jannink S.A., de Jong H., Hessels D., Smit F.P., Melchers W.J. (2016). Detection of High-grade Prostate Cancer Using a Urinary Molecular Biomarker-Based Risk Score. Eur. Urol..

[59] Hendriks R.J., van der Leest M.M.G., Israël B., Hannink G., YantiSetiasti A., Cornel E.B., Hulsbergen-van de Kaa C.A., Klaver O.S., Sedelaar J.P.M., Van Criekinge W. (2021). Clinical use of the SelectMDx urinary-biomarker test with or without mpMRI in prostate cancer diagnosis: A prospective, multicenter study in biopsy-naïve men. Prostate Cancer Prostatic Dis..

[60] Roumiguié M., Ploussard G., Nogueira L., Bruguière E., Meyrignac O., Lesourd M., Péricart S., Malavaud B. (2020). Independent Evaluation of the Respective Predictive Values for High-Grade Prostate Cancer of Clinical Information and RNA Biomarkers after Upfront MRI and Image-Guided Biopsies. Cancers.

[61] Dang X.T.T., Kavishka J.M., Zhang D.X., Pirisinu M., Le M.T.N. (2020). Extracellular Vesicles as an Efficient and Versatile System for Drug Delivery. Cells.

[62] Donovan M.J., Noerholm M., Bentink S., Belzer S., Skog J., O’Neill V., Cochran J.S., Brown G.A. (2015). A molecular signature of PCA3 and ERG exosomal RNA from non-DRE urine is predictive of initial prostate biopsy result. Prostate Cancer Prostatic Dis..

[63] McKiernan J., Donovan M.J., Margolis E., Partin A., Carter B., Brown G., Torkler P., Noerholm M., Skog J., Shore N. (2018). A Prospective Adaptive Utility Trial to Validate Performance of a Novel Urine Exosome Gene Expression Assay to Predict High-grade Prostate Cancer in Patients with Prostate-specific Antigen 2-10ng/ml at Initial Biopsy. Eur. Urol..

[64] Nordström T., Engel J.C., Bergman M., Egevad L., Aly M., Eklund M., Palsdottir T., Grönberg H. (2021). Identifying Prostate Cancer Among Men with Lower Urinary Tract Symptoms. Eur. Urol. Open Sci..

[65] Eggener S.E., Rumble R.B., Armstrong A.J., Morgan T.M., Crispino T., Cornford P., van der Kwast T., Grignon D.J., Rai A.J., Agarwal N. (2020). Molecular Biomarkers in Localized Prostate Cancer: ASCO Guideline. J. Clin. Oncol..

[66] Mohler J.L., Antonarakis E.S., Armstrong A.J., D’Amico A.V., Davis B.J., Dorff T., Eastham J.A., Enke C.A., Farrington T.A., Higano C.S. (2019). Prostate cancer, version 2.2019, NCCN Clinical Practice Guideline in Oncology. J. Natl. Compr. Cancer Netw..

[67] Olivier J., Stavrinides V., Kay J., Freeman A., Pye H., Ahmed Z., Carmona Echeverria L., Heavey S., Simmons L.A.M., Kanthabalan A. (2018). Immunohistochemical biomarker validation in highly selective needle biopsy microarrays derived from mpMRI-characterized prostates. Prostate.

[68] Salmasi A., Said J., Shindel A.W., Khoshnoodi P., Felker E.R., Sisk A.E., Grogan T., McCullough D., Bennett J., Bailey H. (2018). A 17-Gene Genomic Prostate Score Assay Provides Independent Information on Adverse Pathology in the Setting of Combined Multiparametric Magnetic Resonance Imaging Fusion Targeted and Systematic Prostate Biopsy. J. Urol..

[69] Leapman M.S., Westphalen A.C., Ameli N., Lawrence H.J., Febbo P.G., Cooperberg M.R., Carroll P.R. (2017). Association between a 17-gene genomic prostate score and multi-parametric prostate MRI in men with low and intermediate risk prostate cancer (PCa). PLoS ONE.

[70] Associazione Italiana Oncologia Medica (AIOM) Linee Guida CARCINOMA DELLA PROSTATA Edizione 2024 Aggiornamento. 2 Ottobre 2024. https://www.aiom.it/linee-guida-aiom-2024-carcinoma-della-prostata/.

[71] Jairath N.K., Dal Pra A., Vince R., Dess R.T., Jackson W.C., Tosoian J.J., McBride S.M., Zhao S.G., Berlin A., Mahal B.A. (2021). A Systematic Review of the Evidence for the Decipher Genomic Classifier in Prostate Cancer. Eur. Urol..

[72] Erho N., Crisan A., Vergara I.A., Mitra A.P., Ghadessi M., Buerki C., Bergstralh E.J., Kollmeyer T., Fink S., Haddad Z. (2013). Discovery and validation of a prostate cancer genomic classifier that predicts early metastasis following radical prostatectomy. PLoS ONE.

[73] Karnes R.J., Choeurng V., Ross A.E., Schaeffer E.M., Klein E.A., Freedland S.J., Erho N., Yousefi K., Takhar M., Davicioni E. (2018). Validation of a Genomic Risk Classifier to Predict Prostate Cancer-specific Mortality in Men with Adverse Pathologic Features. Eur. Urol..

[74] Spratt D.E., Dai D.L.Y., Den R.B., Troncoso P., Yousefi K., Ross A.E., Schaeffer E.M., Haddad Z., Davicioni E., Mehra R. (2018). Performance of a Prostate Cancer Genomic Classifier in Predicting Metastasis in Men with Prostate-specific Antigen Persistence Postprostatectomy. Eur. Urol..

[75] Zhao S.G., Chang S.L., Spratt D.E., Erho N., Yu M., Ashab H.A., Alshalalfa M., Speers C., Tomlins S.A., Davicioni E. (2016). Development and validation of a 24-gene predictor of response to postoperative radiotherapy in prostate cancer: A matched, retrospective analysis. Lancet Oncol..

[76] Berlin A., Murgic J., Hosni A., Pintilie M., Salcedo A., Fraser M., Kamel-Reid S., Zhang J., Wang Q., Ch’ng C. (2019). Genomic Classifier for Guiding Treatment of Intermediate-Risk Prostate Cancers to Dose-Escalated Image Guided Radiation Therapy Without Hormone Therapy. Int. J. Radiat. Oncol. Biol. Phys..

[77] Nguyen P.L., Huang H.R., Spratt D.E., Davicioni E., Sandler H.M., Shipley W.U., Efstathiou J.A., Simko J.P., Pollack A., Dicker A.P. (2023). Analysis of a Biopsy-Based Genomic Classifier in High-Risk Prostate Cancer: Meta-Analysis of the NRG Oncology/Radiation Therapy Oncology Group 9202, 9413, and 9902 Phase 3 Randomized Trials. Int. J. Radiat. Oncol. Biol. Phys..

[78] Bitting R.L., Wu Y., Somarelli J.A., Proudfoot J.A., Liu Y., Davicioni E., George D.J., Armstrong A.J. (2023). Transcriptomic Signatures Associated With Outcomes in Recurrent Prostate Cancer Treated With Salvage Radiation, Androgen-Deprivation Therapy, and Enzalutamide: Correlative Analysis of the STREAM Trial. JCO Precis. Oncol..

[79] McKay R.R., Feng F.Y., Wang A.Y., Wallis C.J.D., Moses K.A. (2020). Recent Advances in the Management of High-Risk Localized Prostate Cancer: Local Therapy, Systemic Therapy, and Biomarkers to Guide Treatment Decisions. Am. Soc. Clin. Oncol. Educ. Book..

[80] Cullen J., Kuo H.C., Shan J., Lu R., Aboushwareb T., Van Den Eeden S.K. (2020). The 17-Gene Genomic Prostate Score Test as a Predictor of Outcomes in Men with Unfavorable Intermediate Risk Prostate Cancer. Urology.

[81] Helfand B.T., Paterakos M., Wang C.H., Talaty P., Abran J., Bennett J., Hall D.W., Lehman A., Aboushwareb T. (2022). The 17-gene Genomic Prostate Score assay as a predictor of biochemical recurrence in men with intermediate and high-risk prostate cancer. PLoS ONE.

[82] Tward J.D., Schlomm T., Bardot S., Freedland S.J., Lenz L., Cohen T., Stone S., Bishoff J. (2020). Ability of the combined clinical cell-cycle risk score to identify patients that benefit from multi versus single modality therapy in NCCN intermediate and high-risk prostate cancer. J. Clin. Oncol..

[83] Freedland S.J., Gerber L., Reid J., Welbourn W., Tikishvili E., Park J., Younus A., Gutin A., Sangale Z., Lanchbury J.S. (2013). Prognostic utility of cell cycle progression score in men with prostate cancer after primary external beam radiation therapy. Int. J. Radiat. Oncol. Biol. Phys..

[84] Tward J.D., Schlomm T., Bardot S., Canter D.J., Scroggins T., Freedland S.J., Lenz L., Flake DD2nd Cohen T., Brawer M.K., Stone S. (2021). Personalizing Localized Prostate Cancer: Validation of a Combined Clinical Cell-cycle Risk (CCR) Score Threshold for Prognosticating Benefit From Multimodality Therapy. Clin. Genitourin. Cancer.

[85] Tward J., Lenz L., Flake D.D.I.I., Rajamani S., Yonover P., Olsson C., Kapoor D.A., Mantz C., Liauw S.L., Antic T. (2022). The Clinical Cell-Cycle Risk (CCR) Score Is Associated With Metastasis After Radiation Therapy and Provides Guidance on When to Forgo Combined Androgen Deprivation Therapy With Dose-Escalated Radiation. Int. J. Radiat. Oncol. Biol. Phys..

[86] Lenz L., Clegg W., Iliev D., Kasten C.R., Korman H., Morgan T.M., Hafron J., DeHaan A., Olsson C., Tutrone R.F. (2024). Active surveillance selection and 3-year durability in intermediate-risk prostate cancer following genomic testing. Prostate Cancer Prostatic Dis..

[87] Roth J.A., Ramsey S.D., Carlson J.J. (2015). Cost-Effectiveness of a Biopsy-Based 8-Protein Prostate Cancer Prognostic Assay to Optimize Treatment Decision Making in Gleason 3 + 3 and 3 + 4 Early Stage Prostate Cancer. Oncologist.

[88] Tuffaha H., Edmunds K., Fairbairn D., Roberts M.J., Chambers S., Smith D.P., Horvath L., Arora S., Scuffham P. (2024). Guidelines for genetic testing in prostate cancer: A scoping review. Prostate Cancer Prostatic Dis..

[89] Smith C.P., Proudfoot J.A., Boutros P.C., Reiter R.E., Valle L., Rettig M.B., Nickols N.G., Feng F.Y., Nguyen P.L., Nagar H. (2023). Transcriptomic Heterogeneity in High-risk Prostate Cancer and Implications for Extraprostatic Disease at Presentation on Prostate-specific Membrane Antigen Positron Emission Tomography. Eur. Urol. Oncol..

[90] Nikitas J., Subramanian K., Gozal N.B., Ricaurte-Fajardo A., Li E., Proudfoot J.A., Davicioni E., Marciscano A.E., Osborne J.R., Barbieri C.E. (2024). Transcriptomic Profiling of Primary Prostate Cancers and Nonlocalized Disease on Prostate-Specific Membrane Antigen Positron Emission Tomography/Computed Tomography: A Multicenter Retrospective Study. JCO Precis. Oncol..

[91] Kensler K.H., Baichoo S., Pathania S., Rebbeck T.R. (2022). The tumor mutational landscape of BRCA2-deficient primary and metastatic prostate cancer. NPJ Precis. Oncol..

[92] De Bono J., Mateo J., Fizazi K., Saad F., Shore N., Sandhu S., Chi K.N., Sartor O., Agarwal N., Olmos D. (2020). Olaparib for Metastatic Castration-Resistant Prostate Cancer. N. Engl. J. Med..

[93] Smith M.R., Scher H.I., Sandhu S., Efstathiou E., Lara P.N., Yu E.Y., George D.J., Chi K.N., Saad F., Ståhl O. (2022). GALAHAD investigators. Niraparib in patients with metastatic castration-resistant prostate cancer and DNA repair gene defects (GALAHAD): A multicentre, open-label, phase 2 trial. Lancet Oncol..

[94] Abida W., Patnaik A., Campbell D., Shapiro J., Bryce A.H., McDermott R., Sautois B., Vogelzang N.J., Bambury R.M., Voog E. (2020). TRITON2 investigators Rucaparib in Men With Metastatic Castration-Resistant Prostate Cancer Harboring a BRCA1 or BRCA2 Gene Alteration. J. Clin. Oncol..

[95] Nicolosi P., Ledet E., Yang S., Michalski S., Freschi B., O’Leary E., Esplin E.D., Nussbaum R.L., Sartor O. (2019). Prevalence of Germline Variants in Prostate Cancer and Implications for Current Genetic Testing Guidelines. JAMA Oncol..

[96] Abusamra S.M., Solorzano M.A., Quarles J., Luke M., Patel M., Vince R., Jiang R., Volin J., Jacobs M.F., Kaffenberger S. (2024). Detection of Germline Variants in Patients With Localized and Metastatic Prostate Cancer Through Guideline-Based Testing. Urol. Pract..

[97] Carter H.B., Helfand B., Mamawala M., Wu Y., Landis P., Yu H., Wiley K., Na R., Shi Z., Petkewicz J. (2019). Germline Mutations in ATM and BRCA1/2 Are Associated with Grade Reclassification in Men on Active Surveillance for Prostate Cancer. Eur. Urol..

[98] Dias A., Kote-Jarai Z., Mikropoulos C., Eeles R. (2018). Prostate Cancer Germline Variations and Implications for Screening and Treatment. Cold Spring Harb. Perspect. Med..

[99] Leongamornlert D., Mahmud N., Tymrakiewicz M., Saunders E., Dadaev T., Castro E., Goh C., Govindasami K., Guy M., O’Brien L. (2012). Germline BRCA1 mutations increase prostate cancer risk. Br. J. Cancer.

[100] Kote-Jarai Z., Leongamornlert D., Saunders E., Tymrakiewicz M., Castro E., Mahmud N., Guy M., Edwards S., O’Brien L., Sawyer E. (2011). BRCA2 is a moderate penetrance gene contributing to young-onset prostate cancer: Implications for genetic testing in prostate cancer patients. Br. J. Cancer.

[101] Bancroft E.K., Page E.C., Castro E., Lilja H., Vickers A., Sjoberg D., Assel M., Foster C.S., Mitchell G., Drew K. (2014). Targeted prostate cancer screening in BRCA1 and BRCA2 mutation carriers: Results from the initial screening round of the IMPACT study. Eur. Urol..

[102] Castro E., Mikropoulos C., Bancroft E.K., Dadaev T., Goh C., Taylor N., Saunders E., Borley N., Keating D., Page E.C. (2016). The PROFILE Feasibility Study: Targeted Screening of Men With a Family History of Prostate Cancer. Oncologist.

[103] Andreassen C.N., Rosenstein B.S., Kerns S.L., Ostrer H., De Ruysscher D., Cesaretti J.A., Barnett G.C., Dunning A.M., Dorling L., West C.M.L. (2016). Individual patient data meta-analysis shows a significant association between the ATM rs1801516 SNP and toxicity after radiotherapy in 5456 breast and prostate cancer patients. Radiother. Oncol..

[104] Mandel P., Metais P. (1948). Les acides nucléiques du plasma sanguin chez l’homme [Nuclear Acids In Human Blood Plasma]. C R. Seances Soc. Biol. Fil..

[105] Crowley E., Di Nicolantonio F., Loupakis F., Bardelli A. (2013). Liquid biopsy: Monitoring cancer-genetics in the blood. Nat. Rev. Clin. Oncol..

[106] Cheng F., Su L., Qian C. (2016). Circulating tumor DNA: A promising biomarker in the liquid biopsy of cancer. Oncotarget.

[107] Mehra N., Dolling D., Sumanasuriya S., Christova R., Pope L., Carreira S., Seed G., Yuan W., Goodall J., Hall E. (2018). Plasma Cell-free DNA Concentration and Outcomes from Taxane Therapy in Metastatic Castration-resistant Prostate Cancer from Two Phase III Trials (FIRSTANA and PROSELICA). Eur. Urol..

[108] Hennigan S.T., Trostel S.Y., Terrigino N.T., Voznesensky O.S., Schaefer R.J., Whitlock N.C., Wilkinson S., Carrabba N.V., Atway R., Shema S. (2019). Low Abundance of Circulating Tumor DNA in Localized Prostate Cancer. JCO Precis. Oncol..

[109] Bastian P.J., Palapattu G.S., Yegnasubramanian S., Lin X., Rogers C.G., Mangold L.A., Trock B., Eisenberger M., Partin A.W., Nelson W.G. (2007). Prognostic value of preoperative serum cell-free circulating DNA in men with prostate cancer undergoing radical prostatectomy. Clin. Cancer Res..

[110] Chen E., Cario C.L., Leong L., Lopez K., Márquez C.P., Chu C., Li P.S., Oropeza E., Tenggara I., Cowan J. (2021). Cell-free DNA concentration and fragment size as a biomarker for prostate cancer. Sci. Rep..

[111] Fei X., Du X., Gong Y., Liu J., Fan L., Wang J., Wang Y., Zhu Y., Pan J., Dong B. (2023). Early Plasma Circulating Tumor DNA as a Potential Biomarker of Disease Recurrence in Non-metastatic Prostate Cancer. Cancer Res. Treat..

[112] Pope B., Park G., Lau E., Belic J., Lach R., George A., McCoy P., Nguyen A., Grima C., Campbell B. (2024). Ultrasensitive Detection of Circulating Tumour DNA enriches for Patients with a Greater Risk of Recurrence of Clinically Localised Prostate Cancer. Eur. Urol..

[113] Xu L., Mao X., Guo T., Chan P.Y., Shaw G., Hines J., Stankiewicz E., Wang Y., Oliver R.T.D., Ahmad A.S. (2017). The Novel Association of Circulating Tumor Cells and Circulating Megakaryocytes with Prostate Cancer Prognosis. Clin. Cancer Res..

[114] Xu L., Mao X., Grey A., Scandura G., Guo T., Burke E., Marzec J., Abdu S., Stankiewicz E., Davies C.R. (2020). Noninvasive Detection of Clinically Significant Prostate Cancer Using Circulating Tumor Cells. J. Urol..

[115] Al-Hammouri T., Almeida-Magana R., Lawrence R., Duffy T., White L., Burke E., Kudahetti S., Collins J., Rajan P., Berney D. (2023). Protocol for a prospective study evaluating circulating tumour cells status to predict radical prostatectomy treatment failure in localised prostate cancer patients (C-ProMeta-1). BMC Cancer.

[116] Dong L., Hu C., Ma Z., Huang Y., Shelley G., Kuczler M.D., Kim C.J., Witwer K.W., Keller E.T., Amend S.R. (2024). Urinary extracellular vesicle-derived miR-126-3p predicts lymph node invasion in patients with high-risk prostate cancer. Med. Oncol..

[117] McDermott N., Meunier A., Wong S., Buchete V., Marignol L. (2017). Profiling of a panel of radioresistant prostate cancer cells identifies deregulation of key miRNAs. Clin. Transl. Radiat. Oncol..

[118] Bao B., Ahmad A., Kong D., Ali S., Azmi A.S., Li Y., Banerjee S., Padhye S., Sarkar F.H. (2012). Hypoxia induced aggressiveness of prostate cancer cells is linked with deregulated expression of VEGF, IL-6 and miRNAs that are attenuated by CDF. PLoS ONE.

[119] Kachris S., Papadaki C., Rounis K., Tsitoura E., Kokkinaki C., Nikolaou C., Sourvinos G., Mavroudis D. (2021). Circulating miRNAs as Potential Biomarkers in Prostate Cancer Patients Undergoing Radiotherapy. Cancer Manag. Res..

[120] Melton C.A., Freese P., Zhou Y., Shenoy A., Bagaria S., Chang C., Kuo C.C., Scott E., Srinivasan S., Cann G. (2023). A Novel Tissue-Free Method to Estimate Tumor-Derived Cell-Free DNA Quantity Using Tumor Methylation Patterns. Cancers.

[121] Schrag D., Beer T.M., McDonnell CH3rd Nadauld L., Dilaveri C.A., Reid R., Marinac C.R., Chung K.C., Lopatin M., Fung E.T., Klein E.A. (2023). Blood-based tests for multicancer early detection (PATHFINDER): A prospective cohort study. Lancet.

[122] Mahal B.A., Margolis M., Hubbell E., Chen C., Venstrom J.M., Abran J., Kartlitz J.J., Wyatt A.W., Klein E.A. (2024). A Targeted Methylation-Based Multicancer Early Detection Blood Test Preferentially Detects High-Grade Prostate Cancer While Minimizing Overdiagnosis of Indolent Disease. JCO Precis. Oncol..

[123] Eismann L., von Walter P., Jung A., Chaloupka M., Rodler S., Westhofen T., Buchner A., Stief C.G., Stadler T., Schlenker B. (2023). Methylation status of various gene loci in localized prostate cancer: Novel biomarkers for diagnostics and biochemical recurrence. Urol. Oncol..

[124] Liu Q., Reed M., Zhu H., Cheng Y., Almeida J., Fruhbeck G., Ribeiro R., Hu P. (2022). Epigenome-wide DNA methylation and transcriptome profiling of localized and locally advanced prostate cancer: Uncovering new molecular markers. Genomics.

[125] Patel P., Harmon S., Iseman R., Ludkowski O., Auman H., Hawley S., Newcomb L.F., Lin D.W., Nelson P.S., Feng Z. (2023). Artificial Intelligence-Based PTEN Loss Assessment as an Early Predictor of Prostate Cancer Metastasis After Surgery: A Multicenter Retrospective Study. Mod. Pathol..

[126] Shao Y., Bazargani R., Karimi D., Wang J., Fazli L., Goldenberg S.L., Gleave M.E., Black P.C., Bashashati A., Salcudean S. (2024). Prostate Cancer Risk Stratification by Digital Histopathology and Deep Learning. JCO Clin. Cancer Inform..

[127] Pellegrini M. (2023). Accurate prognosis for localized prostate cancer through coherent voting networks with multi-omic and clinical data. Sci. Rep..

[128] Rodrigues N.M., Almeida J.G., Rodrigues A., Vanneschi L., Matos C., Lisitskaya M.V., Uysal A., Silva S., Papanikolaou N. (2024). Deep Learning Features Can Improve Radiomics-Based Prostate Cancer Aggressiveness Prediction. JCO Clin. Cancer Inform..

[129] Tward J.D., Huang H.C., Esteva A., Mohamad O., van der Wal D., Simko J.P., DeVries S., Zhang J., Joun S., Showalter T.N. (2024). Prostate Cancer Risk Stratification in NRG Oncology Phase III Randomized Trials Using Multimodal Deep Learning With Digital Histopathology. JCO Precis. Oncol..

[130] Fay M., Liao R.S., Lone Z.M., Reddy C.A., Muhammad H., Xie C., Jain P., Huang W., Basu H.S., Nair S.S. (2025). Artificial Intelligence-Based Digital Histologic Classifier for Prostate Cancer Risk Stratification: Independent Blinded Validation in Patients Treated With Radical Prostatectomy. JCO Clin. Cancer Inform..

[131] Di Pierro G.B., Salciccia S., Frisenda M., Tufano A., Sciarra A., Scarrone E., Del Giudice F., Asero V., Bevilacqua G., Moriconi M. (2023). Comparison of Four Validated Nomograms (Memorial Sloan Kettering Cancer Center, Briganti 2012, 2017, and 2019) Predicting Lymph Node Invasion in Patients with High-Risk Prostate Cancer Candidates for Radical Prostatectomy and Extended Pelvic Lymph Node Dissection: Clinical Experience and Review of the Literature. Cancers.

[132] Morlacco A., Modonutti D., Motterle G., Martino F., Dal Moro F., Novara G. (2021). Nomograms in Urologic Oncology: Lights and Shadows. J. Clin. Med..

[133] Lorent M., Maalmi H., Tessier P., Supiot S., Dantan E., Foucher Y. (2019). Meta-analysis of predictive models to assess the clinical validity and utility for patient-centered medical decision making: Application to the CAncer of the Prostate Risk Assessment (CAPRA). BMC Med. Inform. Decis. Mak..

[134] Kumar Am S., Rajan P., Alkhamees M., Holley M., Lakshmanan V.K. (2024). Prostate cancer theragnostics biomarkers: An update. Investig. Clin. Urol..

[135] Meijer D., van Leeuwen P.J., Roberts M.J., Siriwardana A.R., Morton A., Yaxley J.W., Samaratunga H., Emmett L., van de Ven P.M., van der Poel H.G. (2021). External Validation and Addition of Prostate-specific Membrane Antigen Positron Emission Tomography to the Most Frequently Used Nomograms for the Prediction of Pelvic Lymph-node Metastases: An International Multicenter Study. Eur. Urol..

[136] Virgo K.S., Rumble R.B., Talcott J.A. (2023). Initial Management of Noncastrate Advanced, Recurrent, or Metastatic Prostate Cancer: ASCO Guideline Update: ASCO Guideline Q and A. JCO Oncol. Pract..

[137] Eastham J.A., Boorjian S.A., Kirkby E. (2022). Clinically Localized Prostate Cancer: AUA/ASTRO Guideline. J. Urol..

[138] Wei J.T., Barocas D., Carlsson S., Coakley F., Eggener S., Etzioni R., Fine S.W., Han M., Kim S.K., Kirkby E. (2023). Early Detection of Prostate Cancer: AUA/SUO Guideline Part II: Considerations for a Prostate Biopsy. J. Urol..

[139] Gillessen S., Bossi A., Davis I.D., de Bono J., Fizazi K., James N.D., Mottet N., Shore N., Small E., Smith M. (2023). Management of Patients with Advanced Prostate Cancer. Part I: Intermediate-/High-risk and Locally Advanced Disease, Biochemical Relapse, and Side Effects of Hormonal Treatment: Report of the Advanced Prostate Cancer Consensus Conference 2022. Eur. Urol..

[140] Salciccia S., Capriotti A.L., Laganà A., Fais S., Logozzi M., De Berardinis E., Busetto G.M., Di Pierro G.B., Ricciuti G.P., Del Giudice F. (2021). Biomarkers in Prostate Cancer Diagnosis: From Current Knowledge to the Role of Metabolomics and Exosomes. Int. J. Mol. Sci..

[141] Wilson T.K., Zishiri O.T. (2024). Prostate Cancer: A Review of Genetics, Current Biomarkers and Personalised Treatments. Cancer Rep..

